# Neural asymmetry in aligning with generous versus selfish descriptive norms in a charitable donation task

**DOI:** 10.1038/s41598-024-55688-0

**Published:** 2024-03-09

**Authors:** Paloma Díaz-Gutiérrez, Christophe Boone, Harshil Vyas, Carolyn H. Declerck

**Affiliations:** Faculty of Business and Economics, University of Antwerp, Antwerp, Belgium

**Keywords:** Psychology, Human behaviour

## Abstract

Social alignment is supported by the brain’s reward system (ventral striatum), presumably because attaining synchrony generates feelings of connectedness. However, this may hold only for aligning with generous others, while aligning with selfishness might threaten social connectedness. We investigated this postulated asymmetry in an incentivized fMRI charitable donation task. Participants decided how much of their endowment to donate to real charities, and how much to keep for themselves. Compared to a baseline condition, donations significantly increased or decreased in function of the presence of descriptive norms. The fMRI data reveal that processing selfish norms (more than generous ones) recruited the amygdala and anterior insula. Aligning with selfish norms correlated on average with reduced activity in the lateral prefrontal cortex (LPFC) and, at the individual level, with decreasing activity in the ventral striatum (VS). Conversely, as participants aligned more with generous norms, they showed increasing activity in the LPFC and, on average, increased activity in the VS. This increase occurred beyond the increased VS activity which was also observed in the baseline condition. Taken together, this suggests that aligning with generosity, while effortful, provides a “warm glow of herding” associated with collective giving, but that aligning with selfishness does not.

## Introduction

Humans, like many social animals, rely on groups for survival and thus care considerably about being included. They often align their thoughts, preferences and behaviors with others to make sure that they fit in. Broadly defined, social alignment can refer to motor synchrony, emotional contagion, or norm compliance, all of which have been shown to increase interpersonal closeness, improve coordination, cohesion, and ultimately group performance and persistence^1^. With so many advantages, it is no surprise that humans are very responsive to cues that facilitate alignment. In this research we will focus specifically on descriptive norms that provide empirical information about what others do, thereby encouraging people to do the same^2^. Descriptive norms can be trivial, guiding people in what clothes to wear or fads to follow, but they can also be more complex with consequences for the well-being of the entire group. For example, people become more generous and will eagerly support charitable organizations when they know of lavish contributions made by others^3^. But they are equally likely to become more selfish, ignoring the underprivileged or evading taxes when they know sufficient others are doing so as well.

Neuroimaging studies have identified a core role of the brain’s reward system in all types of alignment, implying that people align, or “herd,” because it is gratifying and rewarding to be connected with one’s group^1,4^. Research on the neural basis of social alignment, however, has overwhelmingly focused on simple stimuli, such as preferences for faces^5^ or foods^6^ that are of no economic interest, i.e., the decision to align was solely influenced by the information of what others did, irrespective of any other economic or normative concerns. But many, if not most, social situations are more complex, creating a tension between benefits for one-self versus benefits for the group as a whole. In such mixed-motive dilemmas, descriptive norms may conflict with the collectively shared (injunctive) fairness norm. An injunctive norm refers to the generalized notion of what a person “should” do in a particular situation, while a descriptive norm refers to the information of what others actually do^2,7^. When no descriptive norms are available, injunctive norms, which are learned through socialization, assist people in deciding intuitively which behaviors are socially approved and which ones are socially unacceptable^7,8^. While it may be tempting in a mixed motive dilemma to align with a selfish descriptive norm because it is more lucrative, it violates a fundamental normative principle that calls for fairness. Because of this inner conflict with the injunctive norm, aligning with a selfish descriptive norm is less likely to generate the same rewarding “warm glow” we would expect from aligning with a generous descriptive norm. This implies that the neural correlates of aligning with different descriptive norms may not be symmetrical.

Our general hypothesis, that there is a neural asymmetry between aligning with generous- versus selfish norms, derives from a more general theoretical framework proposed by Shamay-Tsoory et al.^1^ that considers the similarities between all different sorts of synchrony and explains how a “warm glow of herding” could come about. The framework distinguishes a neural processing phase from a subsequent (re-)alignment phase. During the processing phase, new information is weighed against one’s own expectations to determine whether or not there is a gap between own behavior and that of others. Neural activity during this phase is expected to engage regions of the salience network (e.g., the anterior insula) to reflect how much one is surprised by information regarding others. Sensing misalignment will (according to the explanatory framework) furthermore recruit executive regions of the brain in order to mend the gap, including the premotor cortex and inferior frontal gyrus (IFG). If the gap between own and other behavior is reduced during the subsequent alignment phase, activation of the brain’s reward system (including the ventromedial prefrontal cortex (VMPFC) and ventral striatum (VS)) reinforces the association between alignment and social connectedness. Thus, people align because they anticipate that it will make them feel good. Even if aligning with generous descriptive norms comes with an economic cost, it will be compensated for by positive affect.

Aligning with selfish descriptive norms, however, is more likely to hamper social connectedness, and would not generate the same positive affect as when aligning with generous norms. More likely, a selfish descriptive norm provides a justification for selfish impulses, implying a different neural mechanism than the one predicted by the theoretical framework proposed in^1^. At the neural level, the cognitive control to restrain self-interest and remain true to a collectively beneficial (fairness) norm has previously been linked to neural activity in the right lateral prefrontal cortex, or rLPFC^9–12^. Conversely, a lack of cognitive control, indicated by attenuated rLPFC involvement, would lead to impulsive decision making and a failure to inhibit self-interest. This is precisely what we expect for aligning with selfish descriptive norms.

**Figure 1:**
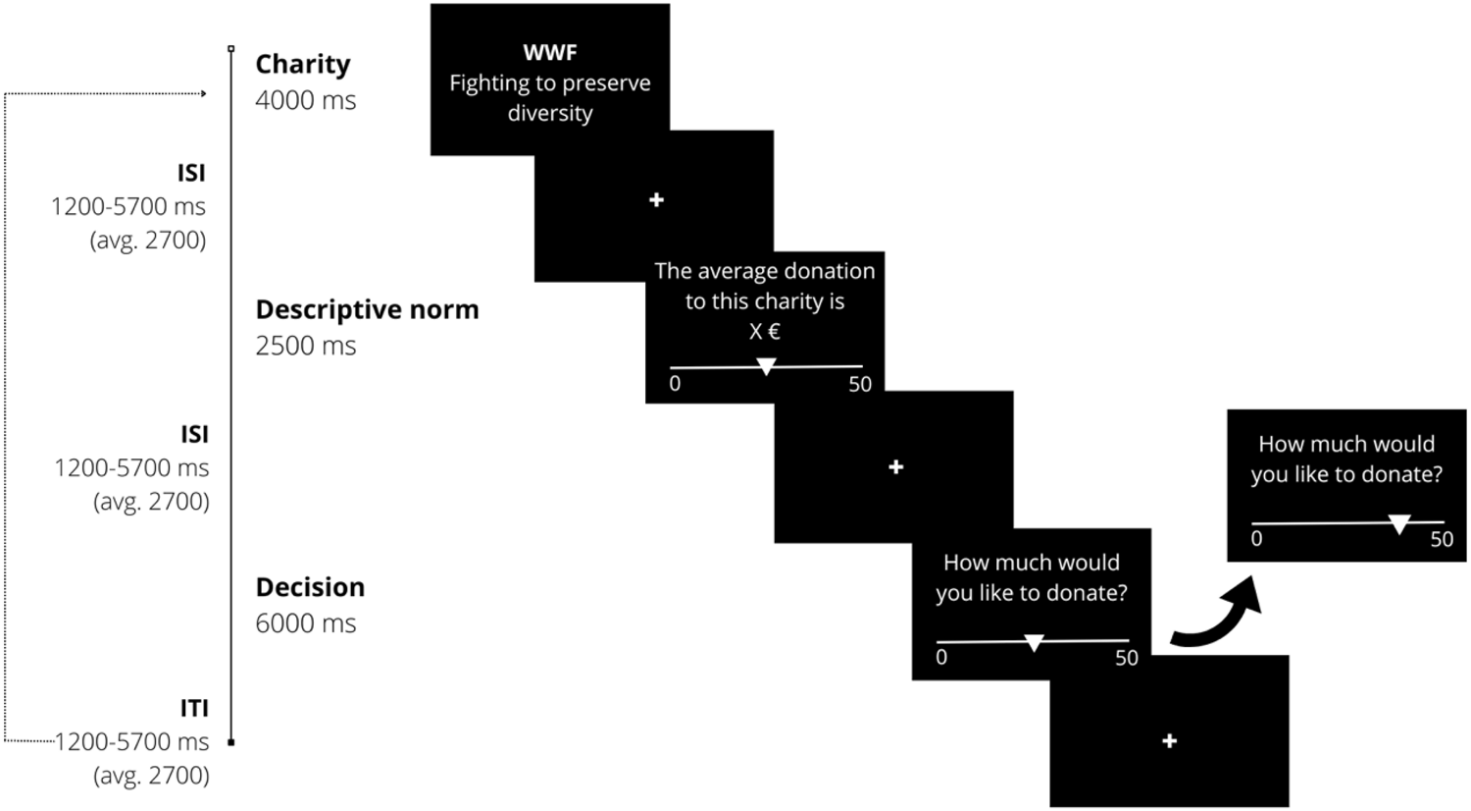
Sequence of events in a single trial. In the first frame (Charity, 4000 ms) participants view a short description of a particular charity. Following a jittered inter-stimulus-interval (ISI; pseudo-logarithmic distribution, mean = 2700 ms; range = 1200–5700), the next frame (Descriptive norm, 2500 ms) gives information on the average donations of others, which varies depending on the experimental condition. In the generous norm condition, X = a randomly chosen value in the range of [32.5–42.5 €]; in the selfish norm condition, the corresponding values for X are in the range of [7.5–17.5 €]; In the no-information condition (shown in figure), X was unspecified. The cursor of the slider is always placed at the average donation of others (in the selfish- and generous norm conditions), or in the middle at 25 € (in the no-information condition). After another jittered ISI, the final frame (Decision, 6000 ms) asks participant to indicate their donation by moving the cursor on the slider.

The current study investigates this postulated neural asymmetry of social alignment by examining the effect of generous versus selfish descriptive norms in a charitable donations task. Participants (N = 50, 10 males, age 18–35) decide how much of a 50 € endowment to donate to a real charity and how much to keep for themselves in 120 trials (see Fig. 1 for the sequence of events in one trial). There are three (within-subject) conditions: in the *generous-* and *selfish norm* conditions participants always receive information of what other people had donated, on average, to that particular charity. This ranged from [32.5–42.5 €] in the generous norm condition, and [7.5–17.5 €] in the selfish norm condition. In the third condition, the average amount donated by others to the charity is unspecified (from now on we call this the *no information* condition for simplicity). However, during these trials an equal split is subtly suggested by anchoring the cursor of a slide ruler at 25 €. Participants can deviate from this suggestion by moving the cursor in order to take more or less for themselves. As taking from charity has previously been established to be a clear incidence of norm violation^13^, the equal share in this condition is considered the injunctive fairness norm shaping participants’ expectations of how they *should* decide when there is no information about what others donate. This condition is necessary as a baseline in order to calibrate the experiment so that “generous” and “selfish” descriptive norms are always relative to the equal split, and not relative to the participant’s own idiosyncratic notion of what fairness entails.

We use functional magnetic resonance imaging (fMRI) to investigate both the processing phase—during which participants receive information that others gave either more than 25 € (a generous descriptive norm) or less than 25 € (a selfish descriptive norm)—and a subsequent alignment (or decision) phase, during which we investigate the neural activity at the time participants actually decide how much to donate in order to align (or not) with the average donations of others. Based on the social alignment model of Shamay-Tsoory et al.^1^ and literature on altruistic decision-making^9–12,14^, we develop specific hypotheses which we test with a functional-based ROI- approach. We rely on previously published work reporting the neural correlates of norm compliance, reward processing, and cognitive control to define specific ROIs (see the methods section for a description of the procedures we used in selecting coordinates). We further justify the functional relevance of these ROIs below as we present the fMRI results. As a robustness check, we repeat all ROI analyses with anatomical masks and report these in the supplementary materials (SI Appendix). While anatomical masks may contain voxels that are not functionally relevant with respect to our hypotheses, the results obtained with this alternative method are in line with those obtained with a functional ROI approach.

## Results

### Effect of descriptive norms on donations

**Figure 2:**
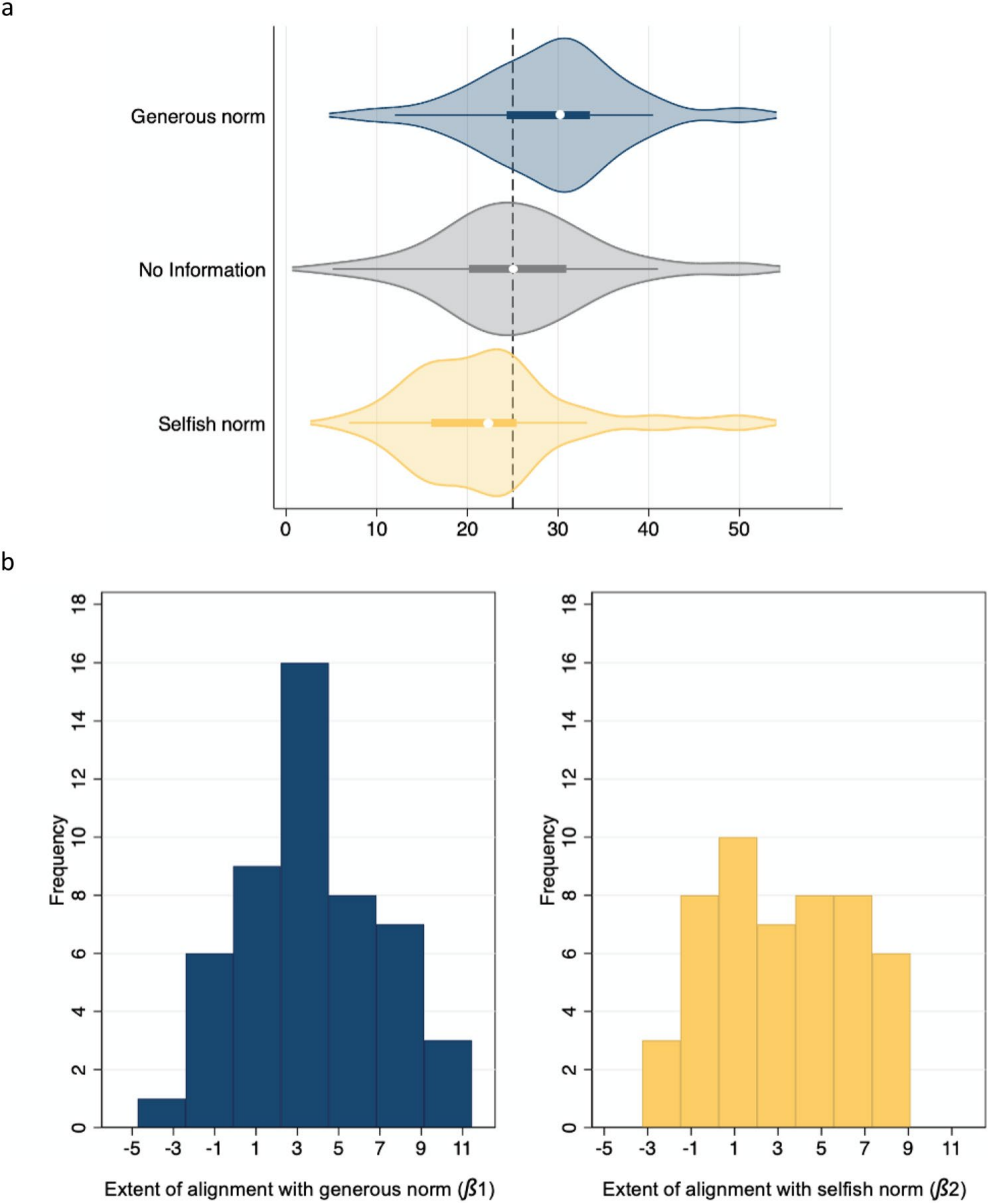
Donations and alignment with descriptive norms. (**a**) Violin plots with the average donation per condition. In the no-information condition participants on average prefer an equal split (*M* = 25.66 €, *SD* = 8.91), and they increase their donations when the norm is generous (*M* = *29.36* €; *SD* = 8.05) while decreasing them after a selfish norm (*M* = 22.54 €; *SD* = 9.012). The vertical dashed line indicates de equal split at 25 €. The white dot represents the median (generous norm = 30.22, no information = 25.07, selfish norm = 22.30). The darkened rectangle represents the first-to-third-interquartile range, while the whiskers demarcate the upper and lower quartiles. (**b**) Frequency distribution of alignment scores to generous and selfish descriptive norms (N = 50). *ß*1 and *ß*2 scores are the coefficients obtained from individual OLS regressions estimating how much each participant aligns with each norm. A Shapiro–Wilk statistic indicates that the distributions of *ß*1 and *ß*2 do no differ significantly from normality (*W* = 0.986, *p* = 0.827; *W* = 0.972, *p* = 0.275, for *ß*1 and *ß*2 respectively).

On average, participants prefer the equal split in the no-information condition (*M* = 25.66 €, *SD* = 8.91), while they donate 3–4 € more when the norm is generous (*M* = *29.36* €; *SD* = 8.05), and roughly the same amount less when the norm is selfish (*M* = 22.54 €; *SD* = 9.012) (Fig. 2a). Fitting a multilevel mixed-effects linear regression model to the panel data (i.e., with random coefficients and intercepts) indicates that the influence of both types of norms on donations is statistically significant and furthermore reveals substantial heterogeneity among individuals, as witnessed by the standard deviations of the estimate of baseline donations (i.e., the constant), as well as of the regression coefficients of generous and selfish norms (Table 1). For comparison purposes, random- and fixed-effects models are included in the supplementary materials, which show very similar coefficients (SI Appendix, Tables S1 and S2). These regression coefficients do not change significantly when controlling for the type of charity (although charities concerning education, health and poverty elicit significantly higher donations compared to social issues, animals, or environment; SI Appendix, Table S1, model 1). The effect of norms on donations is also not dependent on trait-differences in social values, or on trait-measures of conformism assessed two weeks prior to the experiment (SI Appendix, Table S1, models 4 and 6).

**Table 1:** Results of mixed-effect linear regression models.

Predictor	Mixed-effect model
constant	25.659 (1.21)**
Generous norm	3.696 (0.49)**
Selfish norm	− 3.138 (0.48)**
SD Constant	8.417 (0.86)
SD Generous norm	2.704 (0.44)
SD Selfish norm	2.562 (0.44)
χ^2^	2868.05**

N = 5979 observations clustered on 50 individuals (due to missed decision responses, 21 values are missing). The dependent variable is the amount donated (range 0–50 euro), and the independent variables are two dummies indicating whether the presence of the descriptive norm was either generous or selfish. The no-information condition is the omitted category. We report unstandardized coefficients and standard errors in parentheses (SE). The likelihood test (χ^2^) is highly significant, which supports including the random intercept and coefficients in the model. Note also the large standard deviation (SD) of the constant (which estimates the donations in the baseline (no-information) condition) relative to the SD’s of the generous and selfish norm conditions, which hardly differ from one another. **p < 0.001.

To obtain a simple behavioral measure of the extent to which each participant aligns with the norm (which we will use in fMRI analyses), we extract the coefficients of OLS regressions conducted on each individual separately (Eq. 1).

1 $${\text{Donation}}_{{\text{i}}} = {\text{A}}\left( {\text{AVG}}_{{\text{i}}} {\text{No-Information}}\right) + {\beta}1\left( {\text{Generous norm}} \right) + {\beta}2\left( {\text{Selfish norm}} \right),$$

where, the constant A indicates how much the participant donates to charity in the absence of descriptive norms, *ß*1 measures the extent to which the participant aligns with a generous norm, and *ß*2 indicates the extent of aligning with a selfish norm. We reversed the sign of *ß*2 for ease of interpretation. The larger *ß*1 and *ß*2, the more a given participant deviates from his/her baseline donation rate (A) to align with the norm. Consistent with our proposition that alignment to selfishness vs. generosity are different decision processes we find that *ß*1 and *ß*2 are not correlated (Spearman’s rho = 0.066, *p* > 0.6, two-tailed). However, the average amount with which subjects align to descriptive norms does not differ (i.e., the coefficients *ß*1 and *ß*2 are not significantly different; paired t-test *ß*1-*ß*2, z = − 1.154, *p* > 0.2). Frequency distributions of *ß*1 and *ß*2 are shown in Fig. 2b. The mean proportion of the variance explained by the norms (R^2^) is 0.138 (SD = 0.117).

To explore the relevance of alignment behavior in the laboratory, following^15^, we use a self-report conformity scale which measures trait-level differences in the extent to which individuals tend to conform in day-to-day life^16^. Although the correlations between trait-conformism and *ß*1 and *ß2*, are not statistically significant (respectively *ρ*_*ß1*_ = 0.056*, ρ*_*ß2*_ = 0.163, *ps* > 0.1), trait-conformism correlates positively and significantly with the proportion of variance explained by both descriptive norms (i.e., with R^2^ obtained from the regression given by Eq. (1), *ρ* = 0.354, *p* = 0.006). Taken together, we conclude that norms are indeed better predictors of donation behavior for participants scoring high on trait conformism, although both high and low conformists align their donation behavior with the same amount on average (i.e., equal effects sizes *ß*1: U_*ß*1_ = 335.5, *p* > 0.6; and *ß*2: U_*ß*2_ = 349, *p* > 0.4).

### Processing phase: detecting selfish norms correlates with amygdala and anterior insula activation

**Figure 3:**
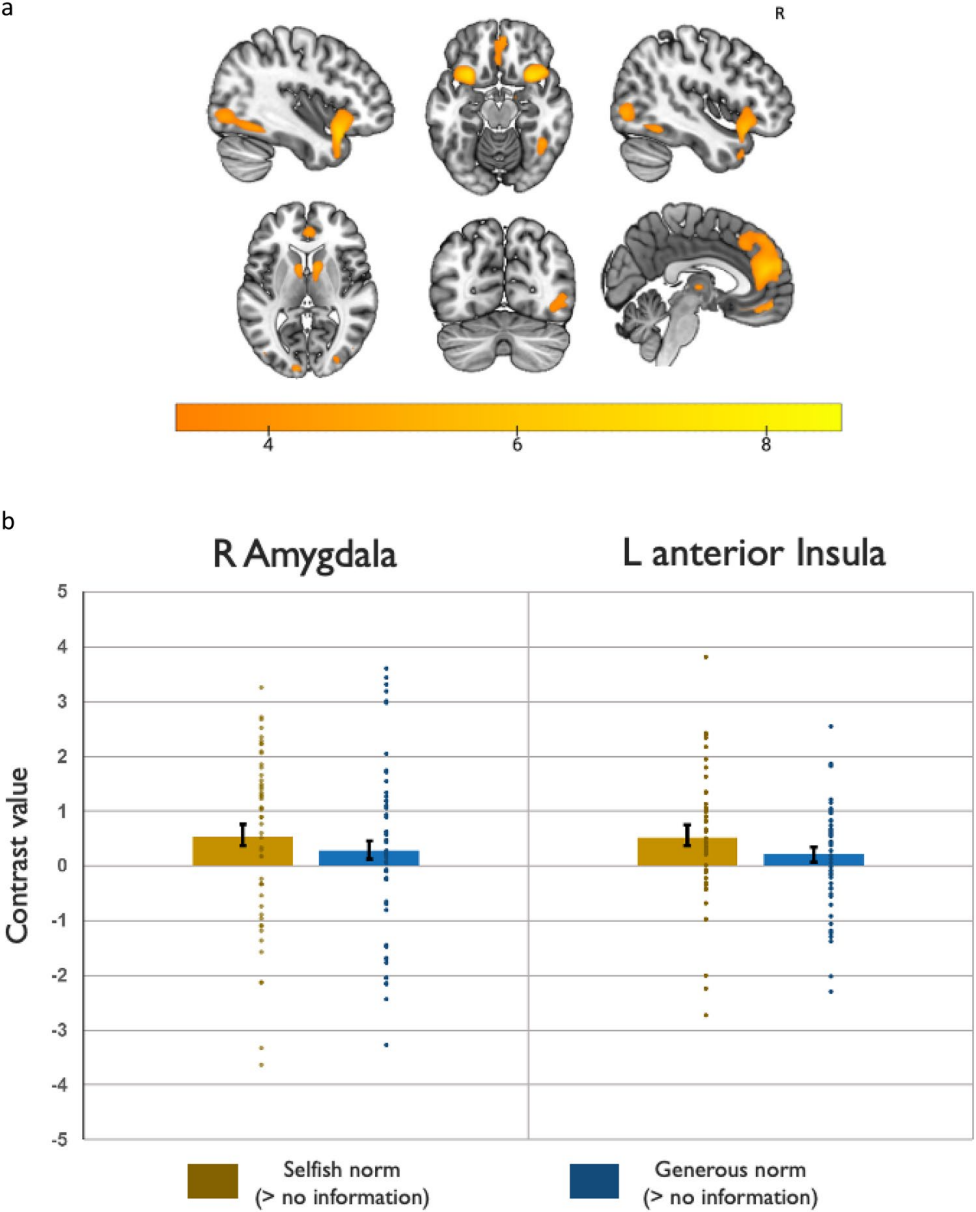
(**a**) Results of a whole-brain analysis showing the effect of processing descriptive norms [generous + selfish norms > no information]. Increased activation is observed in bilateral insula, extending to IFG and temporal poles (k = 556; 33, 16, − 20; k = 439; − 30, 13, − 20), bilateral mPFC (k = 1464; 3, 48, 20; k = 158; − 2, 33, − 23), bilateral caudate (k = 311; 11, 8, 8), and right LOC/fusiform gyrus (k = 339; 48, − 77, 0). Scales reflect peaks of significant t-values (resulting clusters after correcting for multiple comparisons with an FWE cluster-level threshold of *p* < 0.05, obtained from an uncorrected *p* < .001). (**b**) Contrast values in the right amygdala and left aInsula during the processing phase. Peak coordinates are obtained from small volume correction. Contrasts: [selfish norms > no information] and [generous norms > no information]. Error bars indicate the standard error of the mean (S.E.M). Small circles represent individual data points. In both the right amygdala and the left aInsula, the average contrast values for the selfish norm are significantly greater than 0, and also significantly greater than in the generous norm (for the amygdala: Wilcoxon z = 2.003, *p* = 0.023, one-tailed; for the left aInsula (z = 2.254, *p* = 0.012, one-tailed, Bonferroni corrected for comparing 2 ROIs).

An exploratory whole-brain analysis of the processing phase (contrast: [generous + selfish norm > no information]) reveals increased activation in bilateral insula, extending to IFG and temporal poles, as well as bilateral mPFC, bilateral caudate, and right LOC/fusiform gyrus (after FWE-correction thresholded at a 0.05 cluster-level). This widely distributed pattern of activation highlights the saliency of processing the incongruence between the group donation norm and one’s own intended donation, and corroborates the theoretical framework of Shamay-Tsoory et al.^1^ which predicts increased activation in the anterior insula and mPFC to detect a gap between new incoming information and one’s own previously held beliefs, as well as increased activation in IFG to initiate re-alignment in case a gap is detected (Fig. 3a).

We next conduct ROI analyses on the amygdala and the anterior insula (aInsula)—two hubs in the saliency network of the brain specifically involved in emotional processing^17,18^ and implicated in processing fairness violations during money allocation tasks (see^19–23^ for the aInsula and ^24–26^ for the right amygdala). While both generous and selfish descriptive norms are in principle departures of an “equal split” heuristic, only selfishness conflicts with charitable giving and is therefore more likely to elicit an emotional response. Therefore, we specifically hypothesize that especially selfish norms are processed as a breach of the fairness norm, which we test in the left anterior insula and the right amygdala.

While in principle the saliency network includes bilateral insula (which we also find to be activated in the whole brain analysis, as predicted by^1^), we limit the ROI hypothesis testing to the left side because we anticipate that the left aInsula, more than the right aInsula, would represent the conflict between the descriptive and injunctive (fairness) norm and subsequently recruit additional cognitive control to resolve this conflict through social alignment. Substantiating this, only the left aInsula was previously reported to be involved in processing inequity^26^ and conscious error perceptions^27^. A resting-state connectivity analysis has furthermore revealed that the aInsula-prefrontal cortex link is consistent with the left aInsula’s superior role in cognitive control and top-down behavioral modulation^28^. While we purposefully limit the number of ROIs to minimize the constraints imposed by multiple hypotheses tests, for the sake of completeness, we do report analyses with the right anterior insula in the supplementary materials (see SI Appendix, Tables S3a,b).

A focus on the right amygdala is justified because of previous reports identifying it as the primary region activated by unequal outcomes when observing third party money allocations^24,25^. Similarly, a more recent study indicated only right amygdala involvement in processing negative emotions when others take more than a fair share^26^.

We examine the contrasts [selfish norm > no information] and [generous norm > no information] and carry out small volume correction (SVC) and family wise error (FWE) cluster correction at *p* < 0.05 (from an initial voxel-wise forming threshold of *p* < 0.001) on predefined 10-mm spheres around the right amygdala and left aInsula coordinates reported in^26,29^, respectively.

As hypothesized, the contrast [Selfish > No information] shows significantly increased activation in both the right amygdala [peak coordinates: 18, − 7, − 15; pFWE (SVC) = 0.028, k = 2, and left aInsula ([peak coordinates left: − 30, 21, − 3; pFWE (SVC) = 0.031, k = 1]). No significant effects are observed in the contrast [generous norm > no information] for either of these two ROIs. To visualize these effects, we plot the extracted contrast values from the activation peaks from the right amygdala and left aInsula ROIs (Fig. 3b). A Wilcoxon paired tests indicates that the values in [selfish norm > no information] are significantly greater than those in the generous norm condition for both the right amygdala (z = 2.003, *p* = 0.023, one-tailed) and left aInsula (z = 2.254, *p* = 0.012, one-tailed). The pattern is similar and also significant in the right aInsula, SI Appendix, Table S3a). Repeating the analyses with anatomical masks yields the same results (see SI Appendix, Table S3b).

### Decision phase: activation in ventral striatum increases when aligning with generous, but decreases with selfish descriptive norms

The social alignment model presented by^1^ predicts that alignment decisions are driven by anticipation of reward. The two key-regions in reward-based decision-making are the ventromedial prefrontal cortex (VMPFC) and ventral striatum (VS)^30,31^. A meta-analysis on the neural correlates of social conformism furthermore indicates that VS activation increases the more one’s own opinion aligns with that of the group^32^. The hypothesis we test in the current study is whether or not VS and VMPFC activation are *specific* to alignment decisions that are collectively desirable, i.e., generous descriptive norms that do not violate fairness principles. Several previous studies have already demonstrated the involvement of both the VS^31,33^ and VMPFC^31,34^ in charitable donation-type tasks and implicated their activity in generating a “warm glow of giving.”

We conduct two sets of complementary ROI-analyses in both the right (r) and left (l) VS^32^ and in the VMPFC^31^. For the bilateral VS, we use the coordinates reported in the meta-analysis on social conformism^29^; for the VMPFC, we use the coordinates reported in^31^ which specifically related the link between donation behavior and subjective happiness to a smaller region within the VMPFC. With the first, within-subject analysis, we test whether the extent to which participants align with the descriptive norm in each tri*a*l is associated with brain activation in these particular ROIs. With the second, between-subject analysis, we investigate the link between brain activation and participants’ average alignment behavior across all trials (*ß*1 and *ß*2 from Eq. 1).

**Figure 4:**
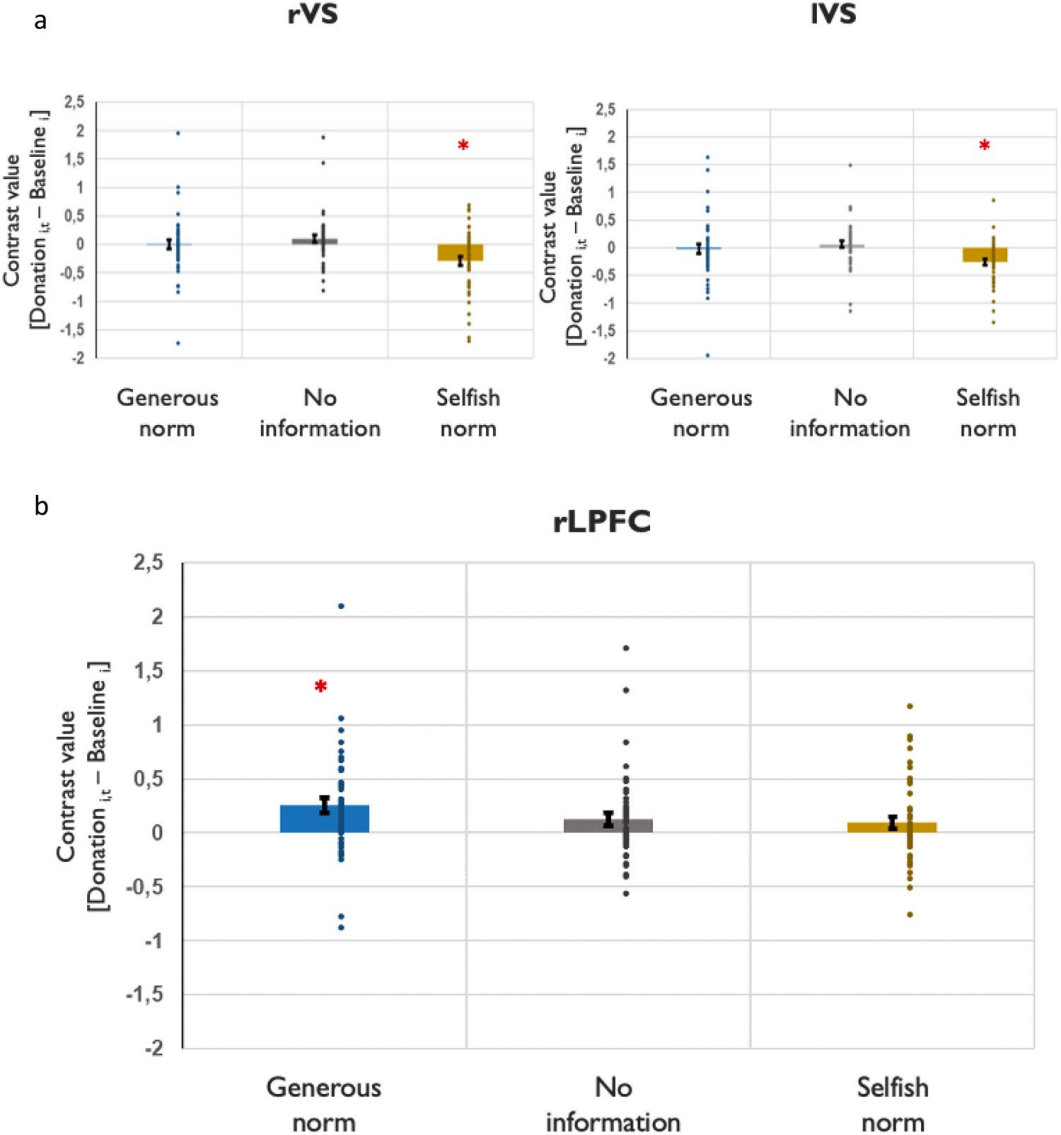
Parametric modulation of the extent of alignment with norms in VS (upper) and rLPFC (bottom). Contrast values testing the effect of the parametric modulator Donation_i,t_ − Baseline_i_ at decision stage during the generous, no information and selfish norm conditions. To further visualize the effects from the small volume correction analysis, we extracted the beta-values from the activation peaks within a 5-mm sphere around the right and left VS, and 10-m sphere around the right LPFC ROIs (initial ROI coordinates derived from Wu et al.^32^ and Spitzer et al.^12^, respectively) and plot them against the effect of the modulation in each condition. Error bars indicate the standard error of the mean (S.E.M). Small circles represent individual data points. (**a**) ROIs = left and right VS. For the selfish norm condition, activation in VS bilaterally decreases as participants donate less than their baseline donation (peak coordinates right: [13, 13, 0] [11 11 − 3]; psFWE (SVC) = 0.009; ks = 1; peak coordinates left: − 12, 16, 5; pFWE (SVC) = .005; k = 9). (**b**) ROI = rLPFC. For the generous norm and no information conditions, activation in rLPFC increases as participants donate more than their baseline donation (peak coordinates: [48, 36, 18]; pFWE (SVC) = 0.008; k = 30).

For the within-subject analysis we conduct SVC on bilateral VS and VMPFC and we include the difference (Donation_i,t_ − A_i_), computed for each trial, as a parametric modulator of the decision phase in the generous and selfish norm conditions separately. This difference score indicates the extent to which a participant deviates from their average decision in the condition without information about what others donated (A_i_). For ease of comparing the size of the deviation in the selfish- and the generous norm condition we reverse the sign of (Donation_i,t_ − A_i_) in the selfish condition. Figure 4a shows that, as participants donate less than A_i_ in the selfish condition, VS activation decreases bilaterally (peak coordinates right: [13, 13, 0], [11 11 − 3]; psFWE (SVC) = 0.009; k = 1; peak coordinates left: [− 12, 16, 5]; pFWE (SVC) = 0.005; k = 9). In the generous condition, aligning with descriptive norms did not show any modulation, neither on VS, nor VMPFC activation. Given that some charities elicit more donations than others (see SI Appendix, Table S1, model1)*,* we run a similar SVC analysis accounting for each trial’s charity type. This additional analysis shows no substantial change in the neural effects of descriptive norms (see SI Appendix, page 5).

Next, we perform between-subjects analyses to test if the average brain activity in these same ROIs, correlates with the extent of alignment with each of the norm conditions. We first compute the contrast values in bilateral VS from the contrast [decision in generous norm condition > decision in no-information condition] and correlate them with the extent to which the participant aligns with generous norms, *ß*1^35^. Note that we do not apply SVC in this case because we are only interested in the correlation, irrespective of whether or not the average brain activation is significantly increased. Consistent with the hypotheses these contrast values correlate positively and significantly with *ß*1 (*ρ* = 0.308, *p* = 0.015 for rVS, *ρ* = 0.275, *p* = 0.025 for lVS). We do the same for the contrast [decision in selfish norm condition > decision in no-information condition]. No significant correlations were found between VS activation and the extent to which the participant aligns with selfish norms, *ß*2 (*ρ* = 0.045 for rVS, *ρ* = 0.119 for lVS). The difference between *ρß*1 and *ρß*2 approaches significance in the rVS (*z* = 1.393, *p* = 0.082, one-tailed). We repeat these analyses with VMPFC but find no noteworthy correlations with *ß*1 nor *ß*2 (see SI Appendix, Table S4a for more details).

Finally, to test if donating to charity also leads to a “warm glow” irrespective of herding (i.e., in the absence of descriptive norms), we extract contrast values in the same ROIs from the “no-information” regressor and compute correlations with A, the individuals’ baseline donation rate. In the rVS the correlation is positive and significant (*ρ* = 0.311,* p* = 0.014, see also SI Appendix, Fig. S1a) meaning that, the more participants donate (on average) in the no information condition, the higher the rVS activation. This result is supported by the parametric modulation analysis showing that, at a more lenient forming threshold of *p* < 0.005, rVS activation increases trial-by-trial as participants donate more than their baseline (A) in the absence of norms (peak coordinates: [8, 16, − 5]; pFWE (SVC) = 0.032, k = 2). Overall, this corroborates previous fMRI experiments reporting a “warm glow of giving” when people donate voluntarily to charity^31,33,36^.

**Figure 5:**
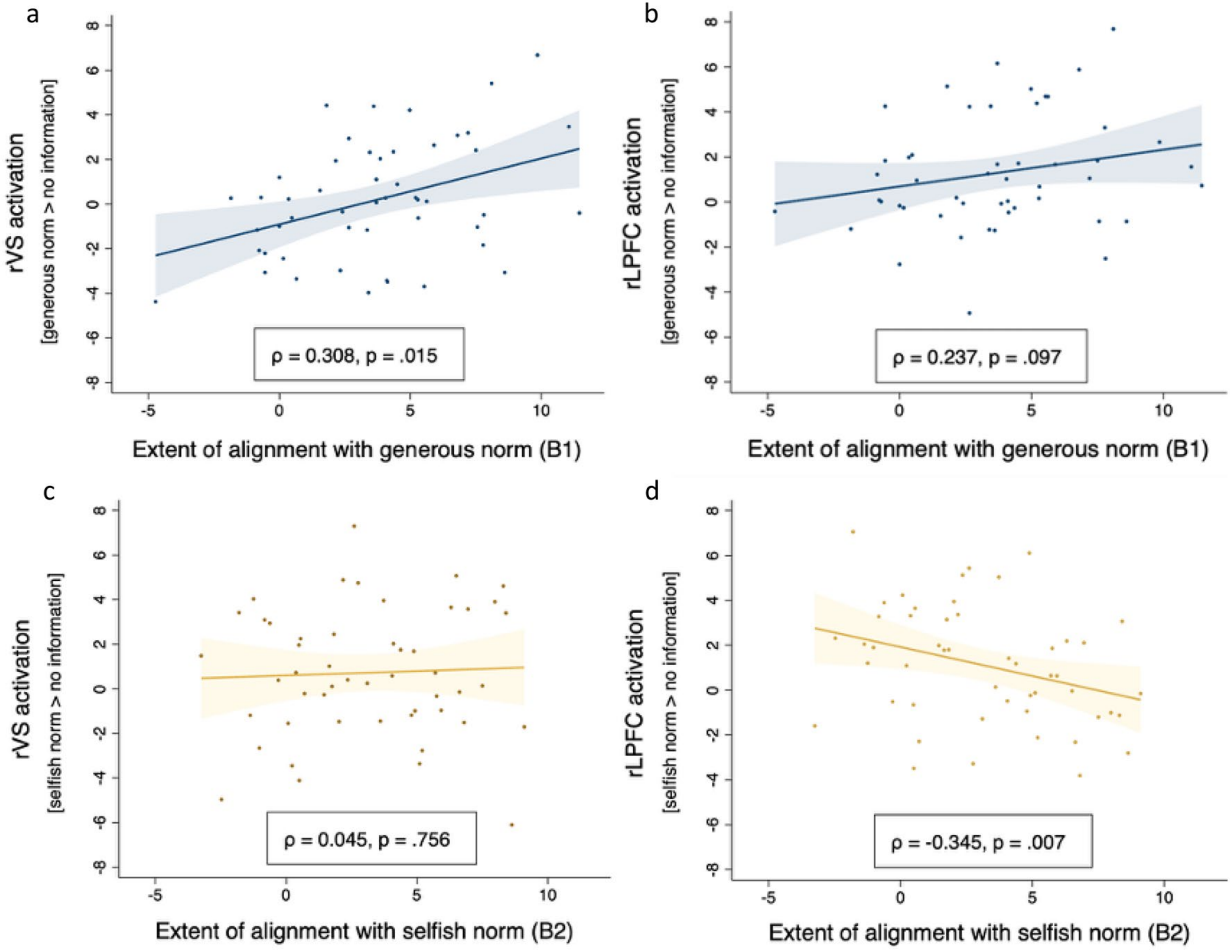
Neural activation in the rVS (left) and rLPFC (right) as a function of individual behavior. The x-axis shows the regression estimates of the extent of aligning with generous or selfish norms (ß1 or ß2); y-axis shows the ROI activation during the decision phase. (**a**) ROI = rVS, generous norm condition. (**b**) ROI = rLPFC, generous norm condition. (**c**) ROI = rVS, selfish norm condition; (**d**) ROI = rLPFC, selfish norm condition. The figure shows that rVS activation correlates positively with generous (a) but does not correlate with selfish norms (c). Activation in rLPFC correlates negatively with alignment to selfish norms (d) and does not correlate with alignment to generous norm (although there is a trend towards positive correlation, b). The colored areas around the fit lines represent 95% CIs (confidence intervals). Results were Bonferroni corrected for multiple comparisons.

Plots relating the contrast values of rVS activation to **ß**1, and** ß**2 are shown in Fig. 5a,c. The positive slopes indicate that, relative to the no information condition, the more participants align with generous norms, the higher the rVS activation. These results suggest that there is a “warm glow” of alignment that adds up to the “warm glow” of voluntary giving when no information of others is available.

### Decision phase: aligning with selfish descriptive norms correlates with decreased cognitive control while aligning with generous increases it

To test the hypothesis that cognitive control is necessary to resist aligning with selfish descriptive norms, we conduct the same analyses as above in the rLPFC, a region which has repeatedly been related to normative and altruistic decision-making in money allocation tasks, both in fMRI^12,14^ and in brain stimulation studies^9–11^. The latter studies have causally related the rLPFC to resisting selfish impulses^10^ and to aligning with internalized fairness goals above and beyond mere rule-following^9^. For this ROI we use the coordinates reported in^12^, which were later also used by^11^.

The trial-by-trial parametric modulation analysis shows that, as an individual is donating more than A_i_ in the generous norm condition, rLPFC activation increases (peak coordinates [48, 36, 18]; pFWE (SVC) = 0.008; k = 30, see Fig. 4b). No effect was found for the selfish norm condition.

The between-subject analysis is consistent with the parametric modulation and also reveals a positive trend in the correlation between activation in rLPFC and the extent to which participants (on average) align with generous norms, *ß*1 (*ρ* = 0.237*, p* = 0.097). More importantly, and corroborating our hypothesis, correlating the contrast value of [decision in selfish norms condition > decision in no information condition] with *ß*2, yields a negative and significant coefficient (*ρ* = − 0.345, *p* = 0.007, Fig. 5d, see SI Appendix, Table S5a for more details). The difference between* ρß*1 *and ρß*2 is significant (z = 3.058, *p* = 0.0011, *one-tailed*).

The link between rLPFC and generous behavior (revealed by the parametric modulation analysis) fits with previous reports showing a causal relation between rLPFC and conforming to costly fairness norms in particular^10^, which suggests that rLPFC may be also recruited to increase one’s baseline donations in order to align with more generous others. Consistently, we find that for a subgroup of participants (N = 12) who are very responsive to generous norms (high*** ß***1), while they resist aligning to selfish norms (low ***ß***2), rLPFC activity is significantly increased during the decision phase, both when the norm is generous (*z* = 2.746, *p* = 0.006) and when it is selfish (*z* = 2.51, *p* = 0.012, see SI Appendix, Fig. S1).

Last, we did not observe significant rLPFC involvement recruitment during donation decisions in the absence of norms, neither in the parametric modulation analysis, nor in the between subject analysis correlating average rLPFC activation and A_i_ (*ρ* = − 0.047*, p* > 0.7*;* see SI Appendix Fig. S2b).

### Robustness check

Overall, we obtain similar results using anatomically-defined ROIs (see SI Appendix, Tables S3b, S4b and S5b). All findings of the norm phase replicate, and so do all findings regarding alignment to generous norms. The effect of aligning to selfish norms is no longer significant in rVS. For the between-subjects analyses, the trends are all in the same direction, but the strength of the correlations is lower.

### Correlations with a priori assessed traits

Group norms may convey different meanings depending on people’s “inner compass.” For example, a selfish descriptive norm that deviates from the injunctive fairness norm may be less of a misalignment to some than to others. We explore if alignment behavior and its underlying neural asymmetry varies according to an individual’s social value orientation (SVO), a stable trait that captures how much a person cares about others during social interactions^37^. Consistently, we find that, in all conditions of the charitable donation task, individuals with a prosocial SVO donate, on average, more than individuals with a proself SVO (*t* = 2.061, *p* = 0.022, one-tailed).

When it comes to alignment decisions, however, we note no behavioral differences between prosocials and proselfs (SI Appendix, Table S1, model 4 and Fig. S3). This is corroborated by the neural data, which indicates no differences in the associated patterns of rVS and rLPFC activation when conducting a between-subject analysis (SI Appendix, Tables S6 and S7). Neither does the parametric modulation analysis reveal any significant activation specific for any of the SVO profiles.

Contrary to prior literature^24^ prosocials do not show increased neural activity in the amygdala when processing selfish norms. Thus, while the SVO construct is validated by the donation data, this cannot be accounted for by differences in processing and aligning to descriptive norms.

## Discussion

The aim of this research was to better understand why humans follow norms. Inspired by a theoretical framework of social alignment^1^ proposing that norm compliance, synchrony, and emotional contagion all share neural correlates that are kin to herding behavior, we investigated with fMRI what happens in the brain when people are confronted with descriptive group norms during a charitable donation task, both when these norms are generous to the charity but costly to the participant, as well as when they are self-serving yet violating an established fairness norm. By including these mixed-motives in the experimental design, we follow up on the call for research testing the generalizability of the theoretical framework proposed in^1^. Our results are mostly consistent with the framework, but also expose some significant differences between aligning with generous- versus selfish descriptive group norms.

In line with the propositions in^1^, we find that norms that deviate from the suggested “equal split” elicit increased activation in the aInsula and medial PFC, associated with detecting the mismatch. Processing this mismatch is also associated with increased activation in the IFG, and premotor cortex to initiate re-alignment. Moreover, the neural data support the predicted asymmetry of aligning with selfish versus generous norms. As predicted, a between-subject analysis shows that, the more a decision aligns with a generous descriptive norm, the more the activation in the rVS of the brain’s reward system increases. This increase in rVS activation is, however, not observed when aligning with selfish norms, and a trial-by-trial parametric modulation analysis furthermore shows that aligning with selfishness is negatively correlated with VS activation. Taken together, these complementary results underscore that aligning with selfishness does not activate the brain’s reward system, whereas systematic alignment with generous behavior does.

Unlike predicted, we do not observe increased activation in the VMPFC with increasing alignment, neither for selfish, nor for generous norms, although several meta-analyses have pointed to an equally important role of the VMPFC in reward-based decision making^30,38^. While we cannot completely dismiss a role of the VMPFC in alignment behavior, the two regions may have distinct functions^39^, with especially the VS contributing to the positive affect which emerges from generous behavior^31,40^, although this claim would require further testing and assessments of participants’ subjective feelings.

Importantly, we note that the increment in rVS activation occurring when participants align (on average) with the generous norm exceeds the rVS activation which is also observed when participants are donating in the absence of a descriptive norm, a finding that is consistent with the *warm glow of giving*, or the satisfaction people experience from merely donating to charity^31,33^. Our results therefore suggest that—in the context of charitable donations—herding may lead to a more inclusive warm glow of collective giving that occurs when behavior is aligned towards helping to take care of those in need. At the societal level, this can explain why the synergistic effect of one’s own motivation to donate, along with observing others’ donations, can create a feed-forward spiral of charitable acts, as seen in the success of crowdfunding events, or the recent solidarity waves to support victims of the Ukrainian war or the 2023 earthquake in Turkey and Syria.

Finally, and equally important, we find a significant decrease in rLPFC activation as individuals become more inclined to align with selfish others. This is opposite the direction of rLPFC activation in the generous norm condition (see Fig. 5b), a between-subjects trend that is also supported by the significant parametric modulation analysis (see Fig. 4b) and by the subgroup of individuals who are almost exclusively aligning with generous norms, resisting the selfish ones. This suggests that aligning with generosity (giving more than one’s baseline) is both effortful and rewarding, while aligning with selfishness (giving less) is neither.

These results, while significant by statistical conventions, should still be interpreted with care for several reasons. First, the statistical power is limited given the typically small sample sizes imposed by the cost and effort of fMRI studies. We tried to improve the reliability of our results by performing SVC on our hypothesized ROIs (for the norm processing stage and the within-subject analysis) and replicate this functional approach to a large extent with anatomical masks.

Second, the ROIs that we selected for this study are parts of functional networks. Focusing on only one aspect (alignment behavior during charitable donations) ignores that many of these regions are involved in several functions depending on input from other brain systems, which in turn, depends on the decision-making context. The LPFC, for example, is part of an executive control network involving working memory, attention selection, planning, and inhibition^41^. Our decision to focus on the rLPFC’s function in restraining self-interest is based on the well-documented role of the rLPFC in altruistic decision-making^10,11^. However, in different contexts that require more strategizing (e.g., interdependent economic games), the LPFC’s has been positively correlated with selfish decision making^42^. Especially individuals who are by nature inclined towards altruism might need to restrain their prosocial intuition and rely on increased LPFC input to become more calculative and protect themselves from free-riders^43,44^. The modulatory role of context and individual differences on reward-based decision-making is not new^45^; the extent to which alignment decisions are similarly affected, however, still requires a more holistic study of how different neural systems that feed into the VS are interconnected, which should go hand in hand with more fine-grained fMRI analyses (i.e., connectivity and pattern analysis).

A third complicating issue is that, even though participants in the current study aligned their decisions with the norm on average, the heterogeneity is remarkable. We attribute this to the fact that the charitable donation task creates a tension between self- and group-interest, making it more difficult for some individuals to decide which norms (selfish or generous) they want to align with, while the anchor at a 50/50 split (priming the injunctive fairness norm) may inhibit other individuals from aligning altogether. Thus the heterogeneity is not surprising and underscores that not all herding behavior is the same^46^. Future studies should further address the role of individual differences, both as precursors of alignment behaviors (predicting the extent to which alignment will, or will not, occur) as well as their modulatory influence on the neural correlates of alignment. Already our data show that descriptive norms (both selfish and generous) explain a greater portion of the variance in donations among high trait-conformists (see SI Appendix, Table S8), suggesting that this trait may be a precursor to alignment behavior. We also investigated the moderating role of SVO, but found no neural explanation for prosocials’ greater aversion to aligning with selfish norms*.* Alignment, however, could be the result of many motives besides those predicted by SVO, each one with a distinct pattern of neural activation^46,47^. An open question is how, at the neural level, different personalities trade off empirical information with their own notion of what they should do when deciding to align or not.

The neural asymmetry in social alignment for situations with a conflict of interest makes evolutionary sense. The activation of the reward system as a result of aligning with generous (but not selfish) others may be an effective way to fulfill the fundamental human need to belong, while the resulting synchrony is also beneficial for the group. When the group prospers, so will its members. Through numerous social exchanges taking place within groups, humans have come to rely heavily on (indirect) reciprocity^48^. Thus, by aligning with others’ good deeds, individuals invest in their own social security, expecting to be repaid should they ever be in need themselves. If, instead, selfishness were to become a norm, the glue that holds the group together would tend to dissolve^13^, jeopardizing its future existence and all the personal advantages that come with it.

The negative consequences of herding, like extreme risk seeking or mob violence are indeed well-documented. Therefore, the social alignment framework proposed in^1^ and elaborated on in this research merits to be scrutinized in different mixed-motive contexts. For example, important contemporary decisions with substantial collective impact such as whom to vote for, whether or not to mandate vaccination, or what to believe regarding climate change, are prone to herding and unfortunately also highly influenced by misinformation that spread via tabloids or media and infiltrate internet echo chambers and virtual groups. Many people identify readily with such groups and seemingly effortlessly conform to their opinions and beliefs^49–51^. Research should investigate if aligning with misinformation also elicits feelings of reward for those individuals that identify strongly with the source of the misinformation, and, if so, find ways to divert them to either rectify the misinformation or align with different groups to strengthen other group ties^52^. In today’s fragmented society with many competing entities and a preoccupation with individualism and materialism, a critical challenge for society is how to mitigate the strong effect of information that exaggerates (or bends) the truth and justifies selfish and/or hurtful behavior. In that respect the “norm nudges” used by policy makers to steer collectively desirable behavior can backfire when the reference group is not carefully assessed^13^. The findings of the current study provide a plausible explanation for why this could be so, by showing that aligning with selfish norms correlates with diminished cognitive control. However, our findings also offer much hope, because, even if selfishness pays off, establishing *collective selfishness* in our experiment did not yield any additional sense of reward, while *collective generosity* did. This endorses the importance of disseminating the good deeds of humanity, a tactic which has been shown to be fruitful in matching programs of fundraising events^3^. Inspiring people to match the generosity of others works because the warm glow of giving appears to be strengthened when shared.

## Materials and methods

### Participants

Fifty volunteers were recruited through social media and e-mail invitations sent to university students (ages 18–35, M = 24.16, SD = 3.88, 10 males). All were right-handed with normal or corrected-to-normal vision, clinically healthy, and complied with MRI safety requirements. Participation was incentivized with a 30 € remuneration and an additional bonus (20 € max) based on answers given during the experimental task. Procedures accorded with the Declaration of Helsinki and were approved by the Commission for Medical Ethics at the University Hospital Ghent (trademark BC-09546). All participants signed an informed consent. Prior to participation we obtained measures of trait-conformism^16^ and SVO^37^ via an online questionnaire. Conformism was assessed with 11 items scored on a 9-point scale (− 4 to + 4). Cronbach alpha was 0.69, which is comparable to reports from other studies^15^. SVO was based on the triple dominance method and used preselect participants so as to balance the final sample so as to have 25 prosocials and 25 proselfs (see *SI Appendix, Supplementary Methods*).

### Experimental task and stimuli

The actual experimental task (conducted in the MRI scanner) consisted of deciding how much to donate (0–50 €) to a given charity. Based on the results of a pilot study (n = 436) we selected 120 existing charities with an equal number of charities belonging to each of six different categories: animals, education, environment, health, poverty and social issues (see *SI Appendix, Supplementary Methods* for the full list and procedures to select them).

PsychoPy^53^ was used to project the stimuli on a screen behind the scanner, visible to participants through a set of mirrors located on the radiofrequency coil. Participants responded with MRI-compatible button boxes held in each hand. By repeatedly pressing a button they moved a cursor on a slider (depicted on the screen) to the desired position. The left index finger moved the cursor to the left, the right index finger moved it to the right, and the middle finger confirmed the decision.

### Procedure

Prior to scanning participants first familiarized themselves with the list of charities before receiving the instruction for the experiment^34^ (see *SI Appendix, Supplementary Methods, Instructions for participants*)*.* They were informed in truth that, at the end of the experiment, one trial from each block would be randomly selected and they would receive a bonus of 40% of the averaged amount from such trials that they decided not to donate. For instance, if they donated 15 € to charity (averaged across those 5 trials), they would receive 40% of the 35 € they chose to keep, that is, 14 €. Then, one trial was picked from those 5, the amount allocated to the charity of the randomly selected trial would be donated in its entirety to the focal charity.

There were 9 practice trials conducted on a laptop (displaying charities that would not be encountered again during the experiment). In the scanner 120 trials were arranged in 5 scanning runs (24 trials/run). The order of the charities was randomized across participants. The sequence of events comprising one trial is depicted and explained in full in Fig. 1. To control for potential motor confounds of moving the slider, the orientation of the scale changed across trials. Thus, in 50% of trials in each run, the scale was oriented from left to right (0 on the left; 50 on the right) and on the other half, it was the other way around.

### Image acquisition and processing

Data were collected on a 3T Magnetom Trio MRI scanner located at UZGent in Ghent, Belgium. T1 weighted anatomical images were acquired with a magnetization-prepared rapid acquisition gradient echo (MP-RAGE) sequence (TR = 2250 ms, TE = 4.18 ms, flip angle = 9°, voxel size = 1 mm^3^). Functional images were obtained with a T2* echo-planar imaging (EPI) sequence (TR = 1730 ms, TE = 30 ms, flip angle = 66°, slice thickness = 2.5 mm, voxel size = 2.5 mm^3^, 50 slices with simultaneous Multi-Slice acquisition). The sequence was divided into 5 runs, consisting of 294 volumes each. MRI images were pre-processed and analyzed with SPM12 software (http://www.fil.ion.ucl.ac.uk/spm/software/spm12). Volumes were realigned and unwrapped (to correct for movement artefacts) and slice-time corrected. Realigned functional images were co-registered with T1, after which they were normalized to MNI space using the parameters from the segmentation of the anatomical image (2.5 mm^3^ voxels, 4th degree B-spline interpolation). Images were smoothed using an 8 mm Gaussian kernel. Low-frequency artefacts were removed using a 128 high-pass filter.

### GLM estimation

We carried out three different General Linear Models in SPM12. A first GLM was estimated to examine the average influence of the norms (analyzing the between subject variance). This model contained, for each run, a regressor for the charity, three regressors corresponding to each of the experimental conditions (generous descriptive norms, selfish descriptive norms, or no information) during the norm processing stage, three regressors for the same conditions during the decision stage, one regressor for trial errors (indicating if a participant missed to confirm their decision or failed to respond at all), and six motion regressors.

Further, we performed a second GLM where we parametrically modulate the difference (Donation_i,t_ − A_i_), where Donation_i,t_ refers to that participant (*i*) donation to that specific trial (*t*) and A_i_ to the average donation made by each participant in the absence of descriptive norms (no information condition). This GLM was carried out to estimate trial-by-trial the modulatory effect of the extent of alignment with the descriptive norm (to analyze the within-subject variance). Here, we included the same regressors as the model above, plus three parametric modulators (PM), one for each decision condition. These PMs indicated how much participants moved away from their baseline donations in each condition in order to align with the descriptive norms. For this reason, in the selfish norm condition the PM is multiplied by (− 1). Because the contrast parameters of the PM cannot be estimated for those individuals who consistently donated the same amount in each trial (or presented very little variance), the sample size for this analysis was reduced to N = 46. In a third GLM, we repeat this analysis where the PMs were calculated with Donation_i,t_ − A_i,c_, where A_i,c_ represents the average donation made to that trial’s specific charity type (see SI Appendix, pages 5, 7). 

In all models, all regressors were convolved with the hemodynamic response function (HRF). The regressors for charity, norm processing phase, and errors were modelled with their entire duration, while the regressors for the decision phase with zero duration. The latter was preferred because we wanted this regressor to estimate the neural activation at the instant of alignment, rather than the process leading to alignment. For this purpose, zero-decision-phases are commonly used in fMRI research^54^.

### ROI definition

To test specific hypotheses, six ROIs were defined a priori based on the criterion that previous studies had demonstrated a clear functional relation with one of the properties underlying social alignment, as defined in the theoretical framework in^1^. These are: left aInsula and right amygdala (for hypotheses regarding the processing of descriptive norms), left and right VS, VMPFC, and right LPFC (for hypotheses regarding alignment decision). The strategy we followed to select independent coordinates for these ROIs was to give priority to meta-analytical coordinates, which we identified by searching the literature using the key terms 'social norms’ in combination with ‘saliency,’ ‘reward,’ and ‘cognitive control.’ This led us to derive the coordinates for bilateral anterior insula, [− 30, 20, 8] and [34, 18, 4], from^29^, who report this region to be consistently activated by norm violations. Note that we formulated only a hypothesis for the left aInsula, but for completeness we included the right aInsula. Coordinates for the bilateral VS, [− 6, 16, 4] and [10, 16, − 2], are retrieved from a meta-analysis^32^ reporting consistent activation of this reward-processing region when conforming to social norms.

For the remaining ROIs for which we could not find appropriate meta-analytical coordinates, we turned to single studies reporting fMRI data obtained in experimental settings that employed money allocation tasks (so-called ‘dictator games’). Similar to the current study, participants in these experiments decided unilaterally how to split an amount of money with another person. The coordinates for the right amygdala, [26, − 4, − 20], are retrieved from^26^ who in turn derived them from the Neurosynth databank (using the key term ‘negative emotion’). This ROI was found to be recruited when processing disadvantageous inequity. For the VMPFC, we retrieved from^31^ the coordinates of a smaller region contained within it, corresponding to the right orbitofrontal cortex, [18, 38, − 17]. The authors linked this particular region to the subjective pleasure of giving. Finally, the coordinates for the right LPFC, [52, 28, 14], are taken from^12^. This particular region was reported to be activated only when participants where allocating money to others in accordance with their own internalized social norm, but not when they were incentivized to donate. The same coordinates were later used in a magnetic stimulation study by^11^ which causally linked activation in this region to restraining selfish impulses in order to allocate money fairly.

### Statistical inference

A whole-brain analysis of the processing phase is performed with a family-wise-error (FWE) correction at a cluster level *p* < 0.05 (from an initial *p* < 0.001 forming threshold) on the contrast [generous + selfish norms > no information].

The ROI analyses pertaining to the processing phase and the within-subject (parametric modulation) analyses during the decision phase are both carried out with small volume correction (FWE cluster corrected at *p* < 0.05, from an initial voxel-wise forming threshold of *p* < 0.001) on predefined 10-mm spheres (5-mm for VS) around the selected coordinates. For the between-subjects ROI analyses of the decision stage, we build spheres around the selected coordinates and extracted the contrast values for each participant with Marsbar. Following recommendations in^55^, we use non-parametric Spearman tests to correlate contrast values to the behavioral variables, which are complemented in the supplementary materials with parametric Pearson tests (SI Appendix, Tables S4–S8) and with Bayesian inference statistics (SI Appendix, Tables S3–S8). The latter evaluate the strength of the evidence relative to the null hypothesis. Results were Bonferroni-corrected for multiple hypotheses testing, i.e., for comparing 2 ROIs during the norm processing phase, and 3 ROIs during alignment.

All ROI analyses were replicated using anatomical masks extracted from the WFU_PickAtlas and the results are reported in the supplementary materials.

### Supplementary Information

Supplementary Information.

## Data Availability

The behavioral dataset generated and analyzed during this study, as well as the fMRI statistical maps are available on OSF (https://osf.io/3tw9m/?view_only=5e3ce04fa8934dfd91aa3083ed6e3a25). Additional analyses and materials are included in the Supplementary Information files. The rest of the data is available from the authors (Paloma.DiazGutierrez@uantwerpen.be or Carolyn.Declerck@uantwerpen.be) upon reasonable request.
