# Supplementary material for: Neural asymmetry in aligning with generous versus selfish descriptive norms in a charitable donation task
Source: Sci Rep. 2024 Mar 9;14:5793. doi: 10.1038/s41598-024-55688-0 (PMC10924952; doi:10.1038/s41598-024-55688-0)
Supplement: Supplementary file 1 — Supplementary Information. [file 41598_2024_55688_MOESM1_ESM.docx]

**Supplementary Information**

**Effect of social norms on donations**

**Table S1. Results of random-effects linear regression model.** N= 5979 observations clustered on 50 individuals. There are 21 missing values due to missed decision responses. The dependent variable is the amount donated (range 0-50 euro), and the independent variables are two dummies indicating whether the presence of the descriptive norm is either generous or selfish. We report unstandardized coefficients and robust standard error (SE) are shown in parentheses. Model 1 shows that charities involving education health and poverty issues elicit higher donations compared to charities involving social issues (the omitted category). However, adding the charities to the regression hardly changes the coefficient of the generous and selfish norm (see main text). Models 2 and 3 add Social Value Orientation (SVO) and corroborate that prosocials (coded 1) donate more than proselfs. Again, the coefficients of the norms do not change much. Model 4 indicates that SVO does not interact with the norms to affect donations. Models 5 and 6 replace SVO with a measure of conformism which neither affects donations directly, nor does it interact with the norms.

| **Predictor** | **Model 1** | **Model 2** | **Model 3** | **Model 4** | **Model 5** | **Model 6** |
| --- | --- | --- | --- | --- | --- | --- |
| constant | **23.021(1.38)**** | **23.299(1.43)**** | **20.662(1.48)**** | **20.605(1.55)**** | **25.006(1.16)**** | **19.629(1.57)**** |
| Generous norm | **3.628(0.49)**** | **3.698(0.49)**** | **3.628(0.49)**** | **3.901(0.66)**** | **3.398(0.49)**** | **3.742(0.54)**** |
| Selfish norm | **-3.186(0.46)**** | **-3.137(0.46)**** | **-3.186(0.46)**** | **-3.330(0.66)**** | **-3.137(0.46)**** | **-3.369(0.50)**** |
| Charity (*Animals*) | **1.631(1.40)** |  | **1.631(1.40)** | **1.654(1.40)** |  | **1.634(1.40)** |
| Charity (*Education*) | **3.456(0.60)**** |  | **3.456(0.60)**** | **3.479(0.60)**** |  | **3.466(0.60)**** |
| Charity (*Environment*) | **0.095(0.96)** |  | **0.095(0.96)** | **0.103(0.96)** |  | **0.094(0.96)** |
| Charity (*Health*) | **6.1187(0.77)**** |  | **6.187(0.77)**** | **6.197(0.77)**** |  | **6.186(0.77)**** |
| Charity (*Poverty*) | **4.711(0.61)**** |  | **4.711(0.61)**** | **4.729(0.61)**** |  | **4.711(0.61)**** |
| SVO |  | **4.721(2.27)*** | **4.719(2.27)*** | **4.805(2.43)*** |  | **5.239(2.29)*** |
| Generous norm*SVO |  |  |  | **-0.545(0.99)** |  |  |
| Selfish norm*SVO |  |  |  | **0.288(0.92)** |  |  |
| Conformity scale |  |  |  |  | **-0.149(0.10)** | **-0.175(0.10)** |
| Generous norm*  Conformity scale |  |  |  |  |  | **0.026(0.04)** |
| Selfish norm* Conformity scale |  |  |  |  |  | **-0.042(0.03)** |
| R^2^ | **0.0729** | **0.0742** | **0.1039** | **0.1041** | **0.0580** | **0.1258** |

*p<.05; **p<.001. *Note*: No-information and social issues are omitted category in norm and charity dummies, respectively.

**Table S2. Results of fixed-effects linear regression model.** N= 5979 observations clustered on 50 individuals. The dependent variable is the donation amount (range 0-50), and the independent variables are two dummies indicating whether the presence of the descriptive norm was either generous or selfish. The no-information condition is the omitted category. We report unstandardized coefficients and standard error in parentheses (SE). **p<.001.

| **Predictor** | **Fixed-effects model** |
| --- | --- |
| constant | **25.669(0.23)**** |
| Generous norm | **3.698(0.32)**** |
| Selfish norm | **-3.137(0.32)**** |
| Within R^2^ | **0.0700** |
| Between R^2^ | **0.0097** |
| Total R^2^ | **0.0432** |

**fMRI Processing phase - Effect of Generous vs. Selfish descriptive norms**

*Whole-brain analysis*

There are no significant clusters of activation when comparing generous vs. selfish norms, in either direction.

*ROI-analysis - functional ROIs*

**Table S3a.** **Contrast between [selfish norm > no information] and [generous norm > no information] during the processing stage for the peak coordinates after small volume correction in right amygdala and bilateral aInsula**. Z-values (one-tailed, for the hypothesis that these two regions would increase their saliency/emotional-related when processing selfish norms) for Wilcoxon tests are indicated with the p-value appearing in parentheses. The two asterisks indicate significance after conducting a Bonferroni correction for multiple comparisons p<.025(0.05/2 t-tests = .025; with our two targeted ROIs right amygdala and left aInsula). Bayes Factor appears below Wilcoxon’s z. Activation in the amygdala and in the aInsula significantly increases when processing selfish norms, in comparison with the increase in the generous norm. The Bayes factor supports with moderate evidence this interpretation for the right aInsula, whereas it supports with weak evidence this interpretation for the left aInsula and right amygdala over the null hypothesis (which would imply no increase in activation).

|  | **amygdala** | **aInsula (l)** | **aInsula (r)** |
| --- | --- | --- | --- |
| **[Selfish norm > no information]**  **> [Generous norm > no information]**  *z* (Wilcoxon signed- rank)  *BF_10_* | *2.003(0.023)***  *1.058* | *2.254(0.012)***  *2.709* | *2.505(0.006)*  *7.685* |

*ROI-analysis - anatomical ROIs*

We replicated the ROI analysis with anatomical masks from the right amygdala and aInsula extracted from the WFU_PickAtlas. Here, the contrast [Selfish > No information] yielded significant increased activation in the amygdala [peak coordinates: 18, -7, -15; pFWE (SVC) = .014, k = 1], and aInsula ([peak coordinates left: -30, 21, -3; pFWE (SVC) = .037, k = 1], [peak coordinates right: 42, 23, -5; pFWE (SVC) = .003, k = 63]). No significant effects were observed in the contrast [generous norm > no information] for amygdala or left aInsula, but it did so for the right aInsula [peak coordinates: 41, 21, -5; pFWE (SVC) = .028, k = 5]). To further examine these effects, we extracted the beta-values from the activation peak within a 10-mm sphere around the amygdala and aInsula peak coordinates, whose initial ROI coordinates were derived from the WFU_PickAtlas plot them, additionally carrying out paired-test between the contrast values in [selfish norm > no information] and [generous norm > no information].

**Table S3b.** **Contrast between [selfish norm > no information] > [generous norm > no information] during the processing stage for the peak coordinates after small volume correction in right amygdala and bilateral aInsula**. Z-values (one-tailed, for the hypothesis that these two regions would increase their saliency/emotional-related when processing selfish norms) for Wilcoxon tests are indicated with the p-value appearing in parentheses. The two asterisks indicate significance after conducting a Bonferroni correction for multiple comparisons of p<.025(0.05/2 t-tests = .025; with our two targeted ROIs right amygdala and left aInsula). Bayes Factor appears below Wilcoxon’s z. Activation in the amygdala and in the aInsula significantly increases when processing selfish norms, in comparison with the increase in the generous norm. The Bayes factor supports with moderate evidence this interpretation for the bilateral aInsula, whereas it supports with weak evidence this interpretation for the right amygdala over the null hypothesis (which would imply no increase in activation).

|  | **amygdala** | **aInsula (l)** | **aInsula (r)** |
| --- | --- | --- | --- |
| **[Selfish norm > no information]**  **> [Generous norm > no information]**  *z* (Wilcoxon signed- rank)  *BF_10_* | *2.003(0.023)**  *1.203* | *2.254(0.012)***  *3.147* | *2.129(0.017)**  *4.006* |

**fMRI Decision phase – Alignment with descriptive norms**

*Role of reward system during alignment with descriptive norms*

*Within-subjects analysis on functionally-defined ROIs*

**Parametric modulation of aligning with the norm controlling for the focal charity type for the ROIs in the reward system.**

As participants donate less than A_i_ in the selfish condition, VS activation decreases in left VS (peak coordinates: -10, 16 ,5; pFWE = .009; k = 1) and right VS with a more lenient forming threshold of p<.005 (peak coordinates: [13, 13, -3]; pFWE =.030; k = 3). In the generous condition, aligning with descriptive norms did not show any modulation on VS or vmPFC activation, neither did giving more than baseline in the no information condition.

*Within-subjects analysis on anatomically-defined ROIS*

**Parametric modulation of aligning with the norm for ROIs in the reward system.**

We replicated the within-subjects analysis with anatomical masks in bilateral VS and vmPFC extracted from the WFU Pick atlas.

In this case, as participants donate less than A_i_ in the selfish condition, activation in the reward system decreases bilaterally, at a more lenient initial threshold of p<.005 ([peak coordinates: -17, 3, -13; pFWE = .028; k = 2], [peak coordinates: -15, 8 , -18; pFWE = .028; k = 2]). In the generous condition, aligning with descriptive norms did not show any modulation on VS or vmPFC activation. However, donating more than during one’s person baseline in the no information condition, only yielded a marginal positive modulation on vmPFC at a more lenient forming threshold of p<0.005 ([peak coordinates: 6, 41, 0; k = 17, pFWE = .056].

*Between-subjects analysis*

**Table S4a.** **Correlation analyses for the ROIs in the reward system with functionally-defined ROIs.** We correlate the contrast values of the brain activation in each ROI during the decision stage in the generous norm condition (> no information condition), in the selfish norm condition (> no information]), and in the no-information condition with ß1, ß2 and A, respectively. Correlation coefficients are given with p-values in parentheses (one-tailed for generous norm and no information conditions, given our hypotheses for rewarding feelings during alignment with collectively desirable decisions and “warm glow” of giving, respectively; two-tailed for selfish norm condition). An asterisk signals statistical significance; two asterisks signal significance after conducting a Bonferroni correction of p<0.016 (0.05/3 t-tests=0.016). Bayes Factors (BF_10_) are computed for each condition and ROI.

|  | **rVMPFC** | **lVS** | **rVS** |
| --- | --- | --- | --- |
| **Correlation with extent of alignment with generous norm (ß1)**  *Spearman’s rho*  *Pearson’s r*  *Bayes Factor (BF_10_)* | 0.009(0.476)  0.078(0.295)  0.285 | 0.279(0.025)*  0.279(0.025)*  2.213 | 0.308(0.015)**  0.388(0.003)**  15.092 |
| **Correlation with extent of alignment with selfish norm (ß2)**  *Spearman’s rho*  *Pearson’s r*  *Bayes Factor (BF_10_)* | 0.144(0.319)  0.099(0.493)  0.222 | 0.119(0.408)  0.140(0.333)  0.278 | 0.045(0.756)  0.045(0.758)  0.185 |
| **Correlation with baseline donations in no information condition (A)**  *Spearman’s rho*  *Pearson’s*  *Bayes Factor (BF_10_)* | 0.255(0.037)*  0.228(0.058)  1.133 | 0.190(0.093)  0.160(0.134)  0.533 | 0.311(0.014)**  0.270(0.029)*  1.968 |

**Table S4b.** **Correlation analyses for the ROIs in the reward system with anatomically-defined ROIs.** We correlate the contrast values of the brain activation in each ROI during the decision stage in the generous norm condition (> no information condition), in the selfish norm condition (> no information]), and in the no-information condition with ß1, ß2 and A, respectively. Correlation coefficients are given with p-values in parentheses (one-tailed for generous norm and no information conditions, given our hypotheses for rewarding feelings during alignment with collectively desirable decisions and “warm glow” of giving, respectively; two-tailed for selfish norm condition). An asterisk signals statistical significance; two asterisks signal significance after conducting a Bonferroni correction of p<0.016 (0.05/3 t-tests=0.016). Bayes Factors (BF_10_) are computed for each condition and ROI.

|  | **rVMPFC** | **lVS** | **rVS** |
| --- | --- | --- | --- |
| **Correlation with extent of alignment with generous norm (ß1)**  *Spearman’s rho*  *Pearson’s r*  *Bayes Factor (BF_10_)* | 0.276(0.026)*  0.297(0.018)*  2.93 | 0.086(0.275)  0.139(0.168)  0.457 | 0.260(0.034)*  0.326(0.010)**  4.647 |
| **Correlation with extent of alignment with selfish norm (ß2)**  *Spearman’s rho*  *Pearson’s r*  *Bayes Factor (BF_10_)* | -0.020(0.892)  0.020(0.889)  0.178 | 0.072(0.617)  0.036(0.807)  0.182 | -0.008(0.958)  -0.092(0.527)  0.214 |
| **Correlation with baseline donations in no information condition (A)**  *Spearman’s rho*  *Pearson’s*  *Bayes Factor (BF_10_)* | 0.132(0.180)  0.211 (0.071)  0.935 | 0.092(0.262)  0.156(0.240)  0.533 | 0.386(0.003)**  0.368(0.004)**  9.995 |

*Role of cognitive control during alignment with selfish descriptive norms*

*Within-subjects analysis on functionally-defined ROIs*

**Parametric modulation of alignment to norms accounting for the focal charity type for the cognitive control-related ROI.**

Here, we do not find any parametric effects on rLPFC during alignment in any condition.

*Within-subjects analysis with anatomical ROIs*

We replicated the within-subjects analysis with anatomical masks in rLPFC extracted from the WFU_PickAtlas.

**Parametric modulation of alignment to norms for the cognitive control-related ROI.**

Here, the trial-by-trial parametric modulation analysis shows that, as an individual is donating more than A_i_ in the generous norm condition, rLPFC activation increases ([peak coordinates: 36, 21, 53; pFWE = .04; k = 33], [peak coordinates: 48, 46, 10; pFWE = .003; k = 153]).

*Between-subjects analysis*

**Table S5a.** **Correlation analyses for the cognitive control-related ROI with functionally-defined ROI.** We correlate the contrast values of the brain activation in rLPFC during the decision stage in the generous norm condition (> no information condition), in the selfish norm condition (> no information]), and in the no-information condition with ß1, ß2 and A, respectively. Correlation coefficients are given with p-values in parentheses (one-tailed for selfish norm condition, given our hypothesis that cognitive control is needed to resist aligning with selfish behavior; two-tailed for generous norm and no information conditions). An asterisk signals statistical significance. Bayes Factors (BF_10_) are computed for each condition and ROI.

|  | **rLPFC** |
| --- | --- |
| **Correlation with extent of alignment with generous norm (ß1)**  *Spearman’s rho*  *Pearson’s r*  *Bayes Factor (BF_10_)* | 0.237(0.097)  0.222(0.120)  0.569 |
| **Correlation with extent of alignment with selfish norm (ß1)**  *Spearman’s rho*  *Pearson’s r*  *Bayes Factor (BF_10_)* | -0.345(0.007)*  -0.324(0.011)*  4.498 |
| **Correlation with baseline donations in no information condition (A)**  *Spearman’s rho*  *Pearson’s r*  *Bayes Factor (BF_10_)* | -0.047(0.746)  -0.035(0.810)  0.181 |

**Table S5b.** **Correlation analyses for the cognitive control-related ROI with anatomically-defined ROI.** We correlate the contrast values of the brain activation in rLPFC during the decision stage in the generous norm condition (> no information condition), in the selfish norm condition (> no information]), and in the no-information condition with ß1, ß2 and A, respectively. Correlation coefficients are given with p-values in parentheses (one-tailed for selfish norm condition, given our hypothesis that cognitive control is needed to resist aligning with selfish behavior; two-tailed for generous norm and no information conditions). Bayes Factors (BF_10_) are computed for each condition and ROI.

|  | **rLPFC** |
| --- | --- |
| **Correlation with extent of alignment with generous norm (ß1)**  *Spearman’s rho*  *Pearson’s r*  *Bayes Factor (BF_10_)* | 0.100(0.490)  0.109(0.451)  0.232 |
| **Correlation with extent of alignment with selfish norm (ß1)**  *Spearman’s rho*  *Pearson’s r*  *Bayes Factor (BF_10_)* | -0.225(0.058)  -0.188(0.095)  0.729 |
| **Correlation with baseline donations in no information condition (A)**  *Spearman’s rho*  *Pearson’s r*  *Bayes Factor (BF_10_)* | -0.048(0.741)  -0.070(0.627)  0.198 |

*Subgroup analysis based on participants’ alignment to norms*

Previous studies have shown a relationship between rLPFC and conforming to costly fair norms (2), which is in line with our observed trend of a positive correlation between activation in rLPFC and alignment with generous norms. To investigate this pattern further, we perform exploratory subgroup analyses based on participants’ actual behavior during the charitable donation task. Based on a median split of the scores of both alignment with generous norms (**ß**1) and alignment with selfish norms (**ß**2), we created 4 groups*: indiscriminate conformers (*high scores in both **ß**1 and **ß**2, n = 13) *nonconformists* (low scores in both **ß**1 and **ß**2, n = 13*), self-interested conformers* (high **ß**1 and low **ß**2, n = 12), and  *generous conformers (*high **ß**2 and low **ß**1; n = 12)*.* For each of these subgroups, we extract from rLPFC the mean contrast values for [decision after generous norm > no information] and [decision after selfish norm > no information] and we test if these contrasts differ from zero. Only the generous conformers show significant increased activation in both the generous ad selfish norm conditions. Next, we compare the level of activation between subgroups, to test whether a particular subgroup presented higher recruitment of rLPFC during alignment with descriptive norms.


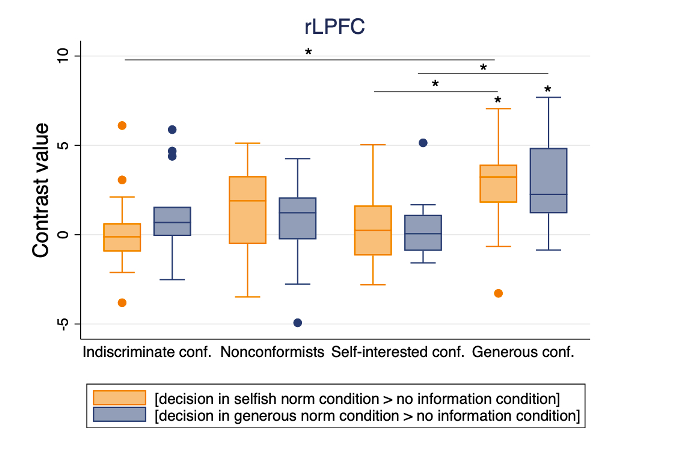


**Figure S1. Subgroup analysis based on alignment with norms in rLPFC.** Figure plots contrast values in rLPFC during decision in the selfish norm condition (> no information condition) and in the generous norm condition (> no information condition) for the different subgroups based on the median split of ß1 and ß2. Only for the generous conformers is the rLPFC significantly increased during the decision phase, both in the generous norm (*z* = 2.746, *p* = 0.006) and selfish norm conditions (*z* = 2.51, *p* = 0.012). Between-groups comparisons with show differences in rLPFC between the generous conformers and self-interested conformers during decisions in both descriptive norm conditions (z =2.367, p =.017 for selfish norm condition; z =2.54, p=.011 for generous norm condition), and between generous conformers and indiscriminate conformers during decisions in the selfish norm condition (z = 2.339, p = .0193). The rest of the comparisons are not statistically significant (p>.07). The line drawn across the box reflects the median, while the whiskers indicate the upper and lower quartiles. Small circles values outside this range.

**
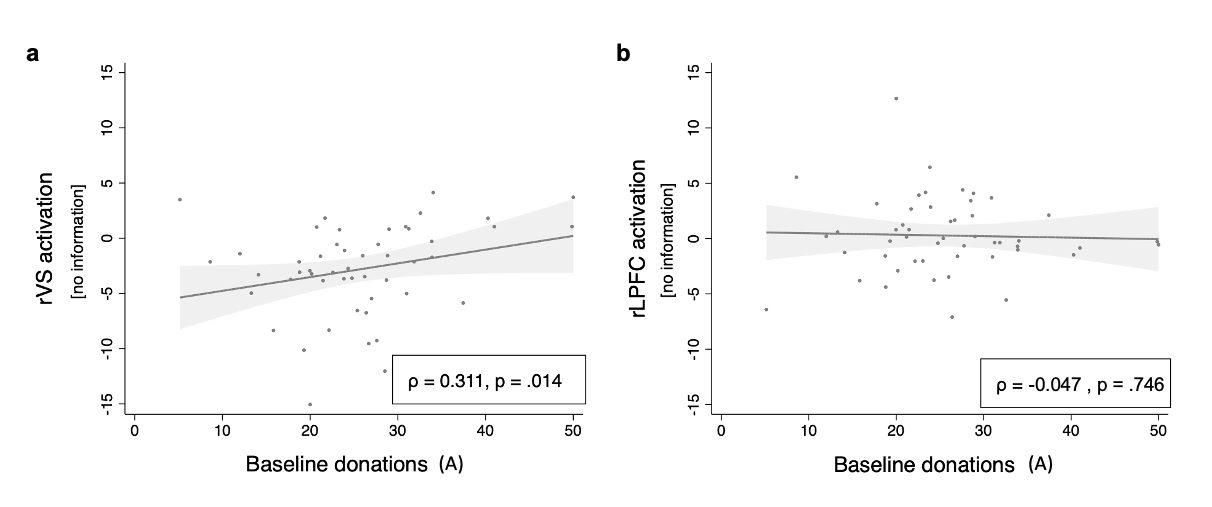
**

**Figure S2. Neural activation in the rVS (left) and rLPFC (right) as a function of baseline donations. a.** x-axis denotes A, the participant’s regression constant indicating their average donation in the no norm condition; y-axis denotes the activation in rVS during the no information condition. The linear regression shows that rVS activation correlates positively with participants’ baseline donations (A), supporting the hypothesis of the “warm glow of giving”. **b.** y-axis denotes the activation in rLPFC during the no information condition. No significant correlation with baseline donations in the no information condition are observed.

**Moderating role of a priori assessed traits in processing and alignment with descriptive norms**

**Social Value Orientation (SVO)**

We further investigate whether SVO, a stable trait that represents people’s preferences towards equality or self-interest, influences the way they align with descriptive norms.


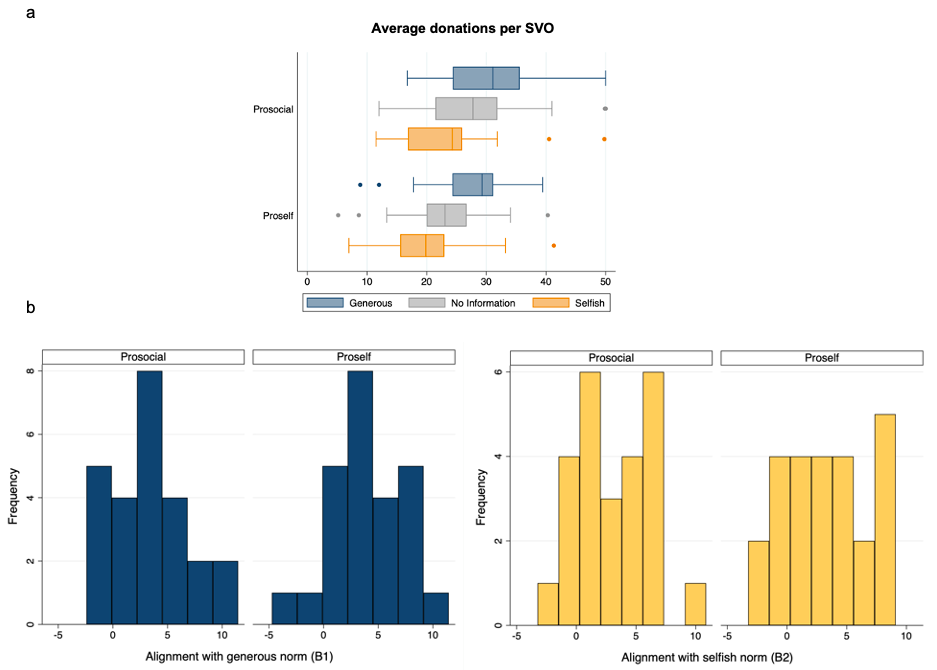


**Figure S3. Relation between SVO and behavior. a.** Average donation per condition and SVO. In general, prosocial participants donated more than proselfs and both increase their donations in the generous norm condition and decrease it in the selfish norm condition. In the no information condition, prosocials donated more than proselfs. The line drawn across the box is the median, while the whiskers demarcate the upper and lower quartiles. Small circles are values outside of this range. **b**. Frequency distribution of alignment scores to generous (ß1) and selfish (ß2) descriptive norms for prosocial and proself participants (N=25 each). *ß*1 and *ß*2 scores are obtained from individual OLS regressions to examine how much each participant align with each norm. Frequency distributions show the high individual variability for *ß*1 and *ß*2, both showing a normal distribution (*W* = 0.938, *p* = 0.130; *W* = 0.975, *p* = 0.772, respectively for prosocials; *W* = 0.977, *p* = 0.815; *W* = 0.948, *p* = 0.229, respectively for proselfs).

Given the individual variability in donations and alignment with norms, we additionally examine the moderating role of SVO on the neural activation during processing and alignment with descriptive norms.

*Processing stage*

Unlike for the whole-sample analysis, we expect that participants will process selfish vs. generous norms depending on whether they have a prosocial or proself SVO. Particularly, we expect that for prosocials, who are inequity averse, the selfish norm (compared to the generous norm) will be more salient and emotionally laden, whereas for proselfs it will not, since it aligns with their intrinsic preferences.

Whole-brain analysis

For prosocials, the comparison of selfish > generous norms ([selfish norm > no information]> [generous norm > no information] yields increased activation in left IFG in an uncorrected map p<.001(k = 148; -42, 38, -5). The opposite contrast does not show any significant clusters. For proselfs, the comparison selfish vs generous norms (in either direction) presents no significant clusters of activation. We also test the between-subject differences in the effects of selfish and generous norms for prosocials > proselfs, and proselfs > prosocials, but neither contrast yields any significant cluster of activation.

ROI- analysis

No significant activation was found with small volume correction in either amygdala or anterior Insula, for any of the contrasts [selfish norms > no information] or [generous norms > no information] for either prosocials or proselfs.

####

#### Decision stage: Role of reward system during alignment with generous descriptive norms

Similar to the rationale for the processing stage, prosocial and proself participants might align with descriptive norms differently. Specifically, given their preference towards equality, prosocials might find alignment with generous norms rewarding, whereas proselfs might not. Therefore, we further investigated the moderating role of SVO in the responses in the reward system during alignment with descriptive norms.

*Within-subjects analysis*

No significant effects were observed with small volume correction in either bilateral VS or VMPFC, for the parametric modulator (Donation_i,t_ – Baseline_i_) in any condition, for either prosocials or proselfs.

*Between-subjects analysis*

**Table S6.** **Correlation analyses for the ROIs in the reward system based on SVO.** Separately for prosocials and proselfs, we correlate the contrast values of the brain activation in each ROI during the decision stage in the generous norm condition (> no information condition), in the selfish norm condition (> no information]), and in the no-information condition with ß1, ß2 and A, respectively. Correlation coefficients are given with p-values in parentheses (one-tailed for generous norm and no information conditions, given our hypotheses for reward during alignment; two-tailed for selfish norm condition). An asterisk signals statistical significance; two asterisks signal significance after conducting a Bonferroni correction of p<0.016 (0.05/3 t-tests=0.016). Bayes Factors (BF_10_) are computed for each condition and ROI. There are no significant correlations between activation in reward-based ROIs and alignment with generous norms for prosocials or proselfs (only weak evidence according to BF). For prosocials, activation in rVMPFC and rVS increase (moderate evidence according to BF) with increasing baseline donations. However, this correlation was significantly different from proselfs’ only in VMPFC (z = 2.21, p = .027; rVS: z = 1.48, p = .139; two-tailed).

|  | **rVMPFC** | | **lVS** | | **rVS** | |
| --- | --- | --- | --- | --- | --- | --- |
|  | *PROSOCIAL* | *PROSELF* | *PROSOCIAL* | *PROSELF* | *PROSOCIAL* | *PROSELF* |
| **Correlation with extent of alignment with generous norm (ß1)**  *Spearman’s rho*  *Pearson’s r*  *Bayes Factor (BF_10_)* | 0.208(0.160)  0.276(0.091)  1.041 | -0.262(0.898)  -0.171(0.794)  0.15 | 0.276(0.091)  0.287(0.082)  1.13 | 0.251(0.113)  0.266(0.100)  0.97 | 0.264(0.101)  0.380(0.030)*  2.53 | 0.254(0.110)  0.395(0.025)*  2.92 |
| **Correlation with extent of alignment with selfish norm (ß2)**  *Spearman’s rho*  *Pearson’s r*  *Bayes Factor (BF_10_)* | 0.095(0.652)  -0.007(0.973)  0.25 | 0.151(0.470)  0.205(0.324)  0.39 | -0.044(0.835)  -0.025(0.904  0.25 | 0.216(0.298)  0.275(0.183)  0.57 | 0.00077(0.999)  -0.015(0.944)  0.25 | 0.064(0.761)  0.076(0.717)  0.26 |
| **Correlation with baseline donations in no information condition (A)**  *Spearman’s rho*  *Pearson’s r*  *Bayes Factor (BF_10_)* | 0.585(0.001)**  0.474(0.008)**  7.355 | 0.005(0.492)  -0.050(0.595)  0.209 | 0.236(0.127)  0.342(0.047)*  1.768 | -0.065(0.624)  -0.162(0.781)  0.150 | 0.491(0.007)**  0.423(0.017)*  3.984 | 0.091(0.333)  0.062(0.383)  0.317 |
|  |  |  |  |  |  |  |

####

#### Decision stage: Role of cognitive control during alignment with selfish descriptive norms

We additionally examine the moderating role of rLPFC in alignment with descriptive norms since proselfs (unlike prosocials), put a higher value on self-interest. This would make it particularly necessary for them to recruit mechanisms of cognitive control to resist aligning with selfish norms, which match their inner preferences. Indeed, Table S8 shows significantly decreased rLPFC activation for proselfs, but this does not differ statistically from the decreased trend in prosocials.

*Within-subjects analysis*

No significant effects were observed with small volume correction in either the rLPFC for the parametric modulator (Donation_i,t_ – Baseline_i_) in any condition, for either prosocials or proselfs.

*Between-subjects analysis*

**Table S7.** **Correlation analyses for the cognitive control-related ROI based on SVO.** Separately for prosocials and proselfs, we correlate the neural parameter of the brain activation in rLPFC during the decision stage in the generous norm condition (> no information condition), in the selfish norm condition (> no information]), and in the no-information condition with ß1, ß2 and A, respectively. Correlation coefficients are given with p-values in parentheses (one-tailed for selfish norm condition, given our hypothesis that cognitive control is needed to resist aligning with selfish behavior; two-tailed for generous norm and no information conditions). An asterisk signals statistical significance. Bayes Factors (BF_10_) are computed for each condition and ROI. For both prosocials and proselfs, activation in rLPFC decreases (weak evidence) as participants align more with the selfish norm (ß2). However, the difference between prosocials and proselfs is not significant (z = 0.42, p = .67; two-tailed).

|  | **rLPFC** | |
| --- | --- | --- |
|  | *PROSOCIALS* | *PROSELFS* |
| **Correlation with extent of alignment with generous norm (ß1)**  *Spearman’s rho*  *Pearson’s*  *Bayes Factor (BF_10_)* | 0.330(0.107)  0.344(0.092)  0.952 | 0.158(0.448)  0.156(0.458)  0.32 |
| **Correlation with extent of alignment with selfish norm (ß2)**  *Spearman’s rho*  *Pearson’s*  *Bayes Factor (BF_10_)* | -0.282(0.086)  -0.285(0.083)  1.11 | -0.395(0.026)*  -0.361(0.038)*  2.10 |
| **Correlation with baseline donations in no information condition (A)**  *Spearman’s rho*  *Pearson’s r*  *Bayes Factor (BF_10_)* | -0.072(0.731)  0.017(0.934)  0.249 | 0.010(0.963)  -0.018(0.933)  0.249 |

**Relationship between other trait-level measures and alignment with norms**

**Table S8.** **Relationship between the conformity scale by Mehrabian & Stefl (1995) and alignment with descriptive norms.** Correlation coefficients (one-tailed, given the expectation of a positive correlation between conformity scale and the alignment scores) indicated with the p-value appearing in parentheses. Bayes Factor (BF_10_) is also shown. The table shows how individual values from the conformity scale and alignment with generous (ß1) or selfish norms (ß2) retrieved from OLS regressions but it does so with the proportion of variance explained (R^2^) by both descriptive norms.

|  | **Conformity scale** |
| --- | --- |
| **Correlation with extent of alignment with generous norm (ß1)**  *Spearman rho*  *Pearson*  *Bayes Factor (BF_10_)* | 0.056(0.349)  0.074(0.305)  0.276 |
| **Correlation with extent of alignment with selfish norm (ß2)**  *Spearman rho*  *Pearson*  *Bayes Factor (BF_10_)* | 0.163(0.129)  0.153(0.145)  0.518 |
| **Correlation with variance explained (R^2^)**  *Spearman rho*  *Pearson*  *Bayes Factor (BF_10_)* | 0.354(0.006)*  0.365(0.005)*  9.464 |

**SUPPLEMENTARY METHODS**

**Measurement of individual differences**

During the recruitment phase, participants were pre-screened based on their Social Value Orientation (SVO) and classified into prosocial or proself profile, following the triple dominance measure (4) which consists of a set of choices (9 in total) where participants are asked to decide between three options that affect their own- and another person’s pay-off. In this context, prosocials value the option that results in equal pay-offs for themselves and the other person, whereas proself prefer to either maximize their pay-offs or the differences between theirs and the other person’s pay-offs. SVO was measured minimum one week ahead of the experiment to avoid possible spill-over effects from answering the triple dominance question to the dependent variable (donating to charity). From the whole sample of pre-screened participants, we selected 25 prosocial and 25 proself participants. To maximize the statistical power of this between-group manipulation, we aimed to select with higher priority those participants who scored consistently 9/9 for each SVO type (N = 34), followed by 8/9 (N = 8), 7/9 (N = 4) and 6/9 (N = 4). Additionally, we collected a conformism measure from the Conformism scale (3).

**List of charities employed in the study**

| Name | Purpose | Category |
| --- | --- | --- |
| *Blauwe Wereldketen (Blue World Chain)* | Improving animal welfare and guaranteeing animal protection | Animals |
| *De PoezenBubbel (The Cats Bubble)* | Food bank for pets of people struggling financially | Animals |
| *De Zonnegloed Wild Animal Sanctuary (The Solar Glow Wild Animal Sanctuary)* | Offering shelter to abandoned or abused wild animals | Animals |
| *Dierenartsen Zonder Grenzen (Vets Without Borders)* | Supporting animal care in Africa | Animals |
| *Dierensaiel Ganzeweide (Animal Refuge Ganzeweide)* | Care and wellness refuge for animals in need | Animals |
| *Forrest & Friends* | Refuge for animals rescued from the meat industry | Animals |
| *GAIA* | Fighting against animal cruelty | Animals |
| *Geels Harto voor Dieren (Geels Harto for Animals)* | Providing care and financial support for animals in need | Animals |
| *Help Animals* | Shelter and relocation for abandoned and found animals | Animals |
| *Hestia Helpt Honden (Hestia Helps Dogs)* | Helping abandoned and abused animals | Animals |
| *Het Blauwe Kruis van de Kust (Blue Cross of the Coast)* | Hosting and relocating dogs and cats | Animals |
| *Het dierenthuisje (The Animal House)* | Providing care for old and ill animals | Animals |
| *Natuurhulpcentrum (Nature Rescue Center)* | Shelter for wild animals that are sick, wounded or in need | Animals |
| *Opvangcentrum Vogels en Wilde Dieren (Shelter for Birds and Wild Animals)* | Providing refuge and care for ill wild birds | Animals |
| *Pairi Daiza Foundation* | Securing animal welfare, protection of endangered habitats, research on wild life | Animals |
| *SenordoRescue* | Offering in adoption dogs whose owners can no longer take care of them | Animals |
| *SHIN* | Rescuing stray and abused dogs | Animals |
| *Vogelopvangcentrum Malderen (Bird rescue centre Malderen)* | Rehabilitation and care of wild birds and ill mammals | Animals |
| *Wilde Dieren in Nood (Wild Animals in Need)* | Refuge for wild animals in need and cooperation for animal research | Animals |
| *WWF* | Fighting to preserve biodiversity | Animals |
| *Alehoppa* | Accessible education for children with mobility problems | Education |
| *Aukas* | Providing economical support for education for Cambodian children | Education |
| *Bednet* | Providing education to sick children | Education |
| *Broeiklas (Greenhouse)* | Educational orientation for vulnerable children | Education |
| *De Verhalenweverij (The Story weaving)* | Fostering reading in disadvantaged children | Education |
| *Doekers (Dokers)* | Supporting educational programs for children and youth in underprivileged countries | Education |
| *Eureka Foundation* | Bringing educational opportunities for people with learning disabilities | Education |
| *Het Open Poortje (The Open door)* | Providing advice to families with educational problems | Education |
| *Joy for Kids* | Sending children to school | Education |
| *MeeGaan (Go along)* | Facilitating learning and work opportunities for those who cannot find a job in the regular market | Education |
| *Muziekkapel Koningin Elizabeth (Musical Chapel Queen Elizabeth)* | Offering music training to talented children and youth around the world | Education |
| *Nino Feliz (Happy Child)* | Providing educational and psychosocial resources to children in poverty | Education |
| *Responsible Young Drivers* | Stimulating youth to drive responsibly | Education |
| *Slim* | Guiding disadvantaged children with their homework | Education |
| *Toekomst Atelier (Future Atelier)* | Offering a learning net to socially vulnerable adolescents | Education |
| *Tonuso* | Training and education for children and youth with difficult family situations | Education |
| *Uilenspel (Owl Game)* | Offering homework orientation for vulnerable children in Gent | Education |
| *Wablieft* | Improving communication and lecture | Education |
| *Waw Make it Work* | Training youth with previous issues with justice to find a job | Education |
| *XaviIndie* | Supporting children education in India to eradicate child work | Education |
| *BE Planet* | Supporting local initiatives that have a positive impact on environment and future generations | Environment |
| *BOS+* | Forest conservation, better and more forest | Environment |
| *Bureau for Conservation and Development* | Promoting sustainable use of natural renewable resources worldwide | Environment |
| *Client Earth* | Holding accountable governments and companies for climate change, nature loss and pollution | Environment |
| *Climate Action Network* | Promoting sustainable climate, energy and development policies throughout Europe | Environment |
| *Collective in Support of Fish workers* | Supporting network of fish mongers | Environment |
| *Cyclists Federation* | Promoting the use of the bicycle as an alternative form of transport | Environment |
| *De Groene Dag (The Green Day)* | Training for a sustainable lifestyle | Environment |
| *Federation for Transport & Environment* | Promoting environmentally conscious transport | Environment |
| *Friends of the Earth* | Environmental protection and sustainable development | Environment |
| *Greenpeace* | Sensibilization, mobilization and action to save and protect the environment | Environment |
| *Grenzeloze Schelde (Boundless Scheldt)* | Improving and restoring the ecosystem of the Scheldt river basin | Environment |
| *HEAL* | Informing and empowering people about the benefits to health of reducing environmental pollution | Environment |
| *Klimaatzaak (Climate case)* | Working to ensure that Belgian authorities follow climate politics | Environment |
| *KNIT IRGT* | Promoting sustainable management of natural resources and development of environment-friendly technologies | Environment |
| *Naturfriends* | Activities for sustainable development of the environment and the society | Environment |
| *Natuurpunt (Nature point)* | Encouraging to do outdoor activities and enjoy natural reserve to protect nature | Environment |
| *Plenty More* | Investing in cooperatives that works for sustainable development | Environment |
| *Polar foundation* | Communicating and educating on polar science and polar research | Environment |
| *River Cleanup* | Cleaning rivers from plastic | Environment |
| *ADO Icarus* | Independence support for handicapped people | Health |
| *Brailleliga (Braille League)* | Helping blind and visually impaired people and supporting eye research | Health |
| *Bring a Smile* | Economic and social support for children with illness | Health |
| *Child Help* | Improving quality of life of spina bifida and hydrocephalus in development countries | Health |
| *De Kolibrie (The Hummingbird)* | Supporting chronic illness patients | Health |
| *DiAwareness* | Funding research and spread awareness on Diabetes | Health |
| *Dokters van de Wereld (Doctors of the World)* | Providing medical care to people without access | Health |
| *Heder* | Providing attention and orientation to handicapped patients | Health |
| *JolieJulie* | Donation of comfortable and fun hats for children who lost their hair | Health |
| *MakeAWish* | Fulfilling the dreams of children with life-threatening conditions | Health |
| *ME TO YOU Foundation* | Contributing to the quality of life of Leukaemia patients | Health |
| *Memisa* | Supporting medical centres and their personnel to provide health care in Africa | Health |
| *Oscare* | Treatment and examining of burnt patients | Health |
| *Pinocchio* | Supporting financially and psychosocially children with burns | Health |
| *Similes* | Supporting care and wellness of people with mental illness and their families | Health |
| *Staf* | Supporting handicapped people to live independently | Health |
| *Think Pink* | Information and funding of breast cancer research | Health |
| *Touw (Rope)* | Offering psychotherapy for people with limited income | Health |
| *twijgje (Twig)* | Offering activities to people psychologically vulnerable | Health |
| *Windroos (Compass rose)* | Developing facilities and activities for people with mental disabilities | Health |
| *ADRA* | Improving quality of life of people living in poverty | Poverty |
| *Amnesty International* | Defence of human rights across the world | Poverty |
| *Armen Tekort (Arms Shortage)* | Connecting people to overcome poverty | Poverty |
| *Beire Cool* | Organizing camp activities for children in poverty | Poverty |
| *BruZelle* | Collection and delivery of sanitary towels for menstruating people with no resources | Poverty |
| *C Dienst (C Service)* | Rehabilitation of homes of people with low income | Poverty |
| *Caritas International* | Providing decent life to crisis victims and people in poverty | Poverty |
| *Cycling out of Poverty* | Giving access to bicycles to people in Africa | Poverty |
| *Feestvarken (Birthday girl)* | Giving a gift and a party box to unprivileged children | Poverty |
| *Join for Water* | Providing infrastructure and the sustainable use of water | Poverty |
| *Leger des Heils (Salvation Army)* | Providing support and solutions to isolation, poverty and exclusion of vulnerable people | Poverty |
| *Netwerk tegen Armoede (Network Against Poverty)* | Working to eradicate poverty and social exclusion | Poverty |
| *Okidoo* | Helping migrant children to be more resilient and manage their emotions | Poverty |
| *Pelicano Foundation* | Offering financial support to children living in poverty | Poverty |
| *Plan International* | Providing opportunities to fight exclusion, vulnerability and gender discrimination | Poverty |
| *Sant Egidio* | Providing home and food to people in need | Poverty |
| *Solidagro* | Increasing the sustainability of food production for economic independency | Poverty |
| *Stichting Wereld Dorpen voor Kinderen (World Villages for Children Foundation)* | Providing education opportunities to children with low income | Poverty |
| *Voedselbanken (Food banks)* | Collecting and distributing food to vulnerable families | Poverty |
| *Welijnszorg (Wellness)* | Supporting projects against poverty | Poverty |
| *Africa Museum* | Protecting biodiversity, culture and natural resources of Africa | Social issues |
| *Binnenste Buiten (Inside Out)* | Social project for vulnerable people | Social issues |
| *BIZON* | Reducing social isolation in vulnerable children and youth | Social issues |
| *Bond zonder Naam (Bond without a Name)* | Contribution to social inclusiveness | Social issues |
| *Cavaria* | Supporting LGTBIQ+ community | Social issues |
| *De Reuzen (The Giants)* | Using stories and imagination to bring people together | Social issues |
| *De Roma* | Organizing festive activities to bring culture over to everyone | Social issues |
| *ECPAT* | Prevention and mobilization to fight sexual exploitation in children | Social issues |
| *Het Scheldeoffensief (The Scheldt Offensive)* | Detecting, defying and showing talent and enthusiasm for theatre of handicapped people | Social issues |
| *HETGEVOLG (Consequence)* | Introducing art to everyone as a place to bring people together | Social issues |
| *Jongerenzorg (Youth Care)* | Offering orientation and support to people living under difficult circumstances so they feel socially valued | Social issues |
| *Knoest (Knot)* | Organizing camp for socially vulnerable youth | Social issues |
| *Kras Jeugdwerk (Scratch Primary)* | Organizing social, sportive and cultural activities for children and youth | Social issues |
| *LEJO* | Empowering socially vulnerable youth | Social issues |
| *MinorNdako* | Guidance and attention to children and their families to achieve social inclusion | Social issues |
| *One Trick Pony* | Laboratory of contemporary music | Social issues |
| *Samen Plannen (Planning Together)* | Fostering connection and social inclusion | Social issues |
| *Studio Globo* | Stimulating and supporting solidarity | Social issues |
| *Women in Film, Television & Media* | Working towards gender and rights equality for women in media | Social issues |
| *YWCA* | Fighting for female rights and empowerment | Social issues |

**Procedure to obtain the final list of charities**

The initial list of charities to which Belgian participants can donate started from a total of 308 candidates, obtained from [Goodgift.be](file:///C:\Users\pdiazgutierrez\Downloads\Goodgift.be\), [Goedgeven.be](https://www.goedgeven.be/) and general search on [Google.com](file:///C:\Users\pdiazgutierrez\Downloads\Google.com). To reduce such list, we prioritized those charities that had national or international outreach, whose purpose was stated unambiguously, and could easily be classified in one of the following 6 categories: animals, education, environment, health, poverty, social issues.

That procedure yielded 130 charities, which were tested in a pilot study with university students. In that pilot (N = 436), we asked participants their intended donations to those charities, as well as how familiar they were with the charity and how close they felt to the charity. Because a very high or low familiarity or closeness score could have a disproportionately large influence on participants’ behavior in the main experiment (overruling any possible modulating effect of descriptive norms), we identified outliers (scores that exceeded +/- 3 SD). Four charities with extreme familiarity scores were removed from the list. An additional 6 charities were removed from the list to come up with a final sample with exactly 20 charities in each of the six categories.

**Instructions for participants**

“Now you are going to make a series of decisions inside the scanner.

In each trial, **you will receive an endowment of 50 eur, and you have to decide how much of those 50 eur you would like to donate to a charity**. You will see in each trial a different charity with their names and purpose (the same from the list you just saw).


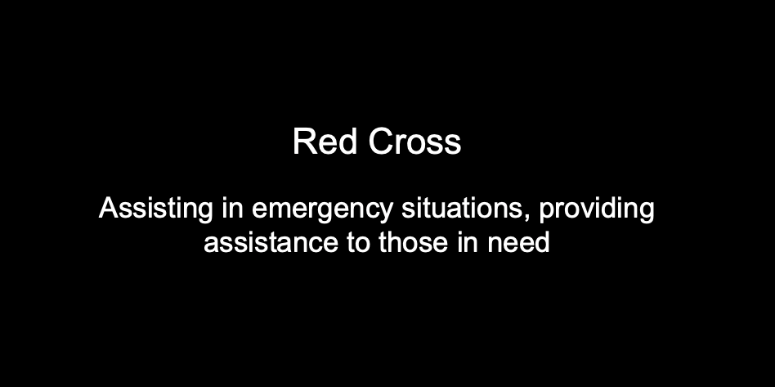


When you finish the experiment, we will select one trial per block, and **you will get 40% of the average amount you decided to keep to yourself in those trials**. Then, we will pick one of those 5 trials, and the **selected charity will receive the donation you made for them**.

After each charity, you will see information about what other people like you decided to donate to that charity, with a slider marking the indicated amount.


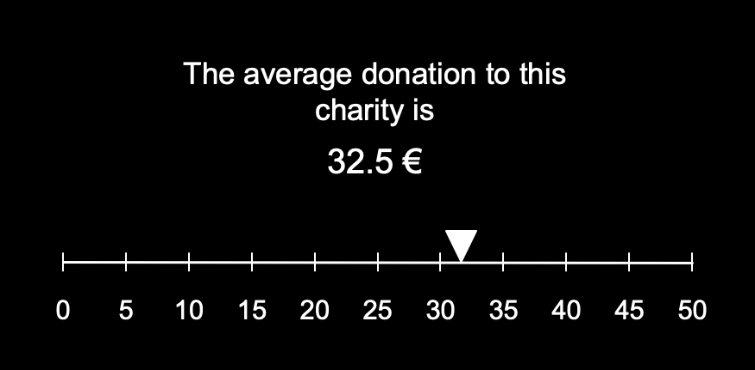


For some charities, we don’t have that information, so you will see “unknown”.


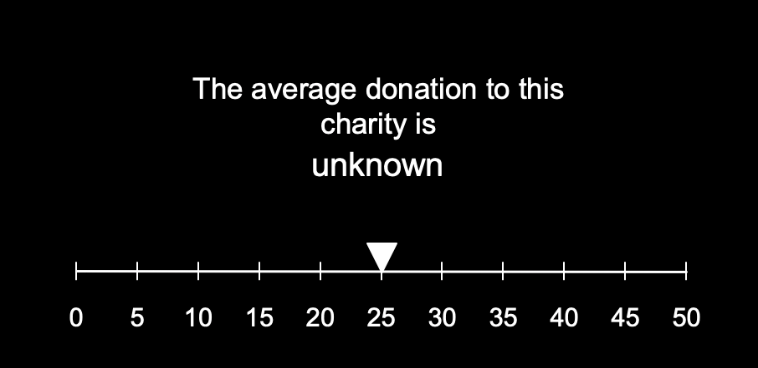


**At that stage, you don’t have to make your decision yet.**

In the next slide, you will see the slider again, this time with a question of **how much would you like to donate**. The marker will be at the same place as before, but you **can freely move the marker until you reach your desired donation**. Once you make your decision, you have **to accept it** with a different button (see below the design and button presses). Importantly, **if you want to donate the amount already indicated by the slider, you have to accept it too**.


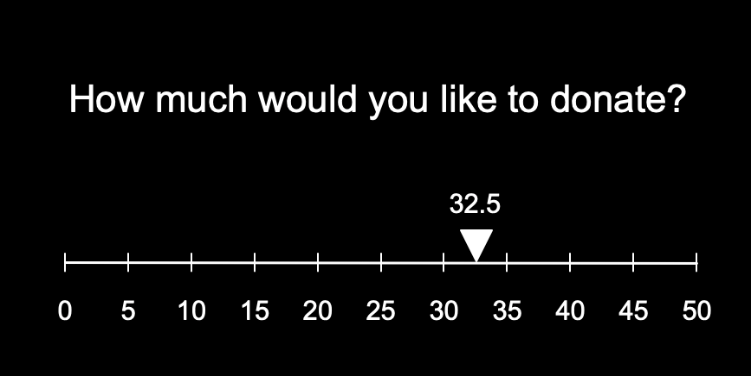


To allow you to move along the scale easily, each press will move the slider 2 eur to the left or right. Note that you have 6 sec to decide, you have time, but remember that you also have to accept the donation.

Importantly, **the scale will change its horizontal orientation from left to right across trials, so pay attention.** Nonetheless, you will always see a number indicating the donation you are choosing at each time.


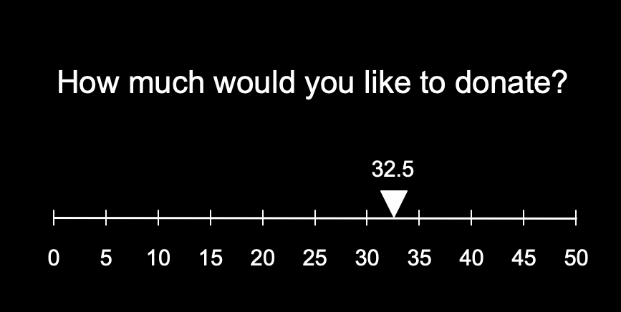

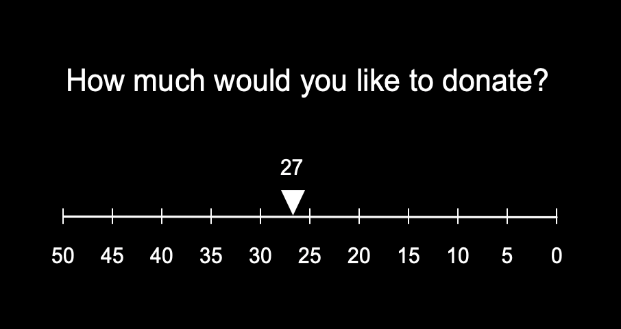


You will perform **5 blocks**, each with different charities, and between blocks, we will stop the scanner and ask you if everything is ok, in case you need to tell us something.

Please, it is very important that **you stay still and don’t cross your arms or legs**. You have an emergency button, so in case you feel uncomfortable or need to stop the experiment, you can press it.

Button presses:

You have two response pads: one from your left and one for your right hand. Use your left (yellow) and right (green) index fingers to move the slider to the left or right respectively. To accept the donation, you will have to press the right middle finger (red).”

**References**

1. B. Diedenhofen, J. Musch, Cocor: A comprehensive solution for the statistical comparison of correlations. *PLoS One* **10**, 1–12 (2015).

2. D. Knoch, A. Pascual-Leone, K. Meyer, V. Treyer, E. Fehr, Diminishing reciprocal fairness by disrupting the right prefrontal cortex. *Science (80-. ).* **314**, 829–832 (2006).

3. A. Mehrabian, C. A. Stefl, Basic Temperament Components of Loneliness, Shyness, and Conformity. *Soc. Behav. Personal. an Int. J.* **23**, 253–264 (1995).

4. P. A. M. Van Lange, The pursuit of joint outcomes and equality in outcomes: An integrative model of social value orientation. *J. Pers. Soc. Psychol.* **77**, 337–349 (1999).
